# METTL3-induced lncRNA GBAP1 promotes hepatocellular carcinoma progression by activating BMP/SMAD pathway

**DOI:** 10.1186/s13062-023-00409-2

**Published:** 2023-09-01

**Authors:** Runkun Liu, Guozhi Yin, Hang Tuo, Yixian Guo, Yifeng Zhu, Lei Zhang, Wei Yang, Qingguang Liu, Yufeng Wang

**Affiliations:** 1 Department of Hepatobiliary Surgery, The First Affiliated Hospital of Xi’an Jiaotong University, Xi’an, 710061 Shaanxi China; 2 Department of Geriatric Surgery, The First Affiliated Hospital of Xi’an Jiaotong University, Xi’an, 710061 Shaanxi China

**Keywords:** Hepatocellular carcinoma, m^6^A, GBAP1, miR-22-3p, BMPR1A

## Abstract

**Background:**

Hepatocellular carcinoma (HCC) is one of the most common and challenging cancers in the world. N6-methyladenosine (m^6^A) modification and long non-coding RNAs (lncRNAs) play critical roles in the progression of HCC. However, there are few reports on genome-wide screening and functional annotations of m^6^A-methylated lncRNAs in HCC.

**Methods:**

The expression levels of m^6^A methyltransferase METTL3 and the association with the prognosis in HCC were determined by RT-qPCR, public dataset platforms. Then, RNA-seq, Pearson correlation analysis, MeRIP-qPCR, RNA half-life assay, gene site-directed mutation, RIP assay and RT-qPCR analysis were employed to determine the downstream target of METTL3 in HCC. Subsequently, the expression levels and roles of lncRNA glucosylceramidase beta pseudogene 1 (GBAP1) in HCC were determined by Kaplan–meier curves, RT-qPCR, in vitro functional experiments and in vivo tumorigenesis and lung metastasis models. Then, the downstream target and pathway of GBAP1 were explored by GO biological process, KEGG pathway enrichment, luciferase reporter assay, RIP assay and rescue experiments and so on.

**Results:**

METTL3 was upregulated in HCC and closely related to HCC prognosis. And METTL3 induced GBAP1 expression by acting as the m^6^A writer of GBAP1 and IGF2BP2 worked as its m^6^A reader. Clinically, GBAP1 expression was significantly associated with tumor size, venous infiltration, TNM stage and prognosis of HCC, Functionally, GBAP1 promoted HCC metastasis and growth both in vitro and in vivo. Furthermore, GBAP1 acted as the molecular sponge for miR-22-3p to increase the expression of bone morphogenetic protein receptor type 1A (BMPR1A), which then activated BMP/SMAD pathway in HCC cells.

**Conclusions:**

Our findings demonstrated that METTL3-induced GBAP1 promoted migration, invasion and proliferation of HCC cells via GBAP1/miR-22-3p/BMPR1A/SMAD axis. GBAP1 could be a potential prognosis indicator and therapeutic target for HCC.

**Supplementary Information:**

The online version contains supplementary material available at 10.1186/s13062-023-00409-2.

## Background

Hepatocellular carcinoma (HCC) is one of the most common cancers in the world, with the characteristics of high morbidity and mortality [1] Though the advances in HCC therapy have been made in the last decades, the general outcome of HCC patients remain not optimistic [1]. Therefore, it is urgent for us to further elucidate the etiology and exact molecular mechanisms of HCC, and discover some new and practical therapeutic strategies for HCC.

N6-Methyladenosine (m^6^A) methylation is one of the most abundant and most well-studied epitranscriptomic modifications in RNA [2]. As an m^6^A writer, METTL3 is a critical mRNA methyltransferase [2]. Besides mRNA, more and more non-coding RNAs, such as microRNA (miRNA), long noncoding RNA (lncRNA) and circular RNA (circRNA), have been reported to be modulated by m^6^A modification [3–5]. Abnormal levels of m^6^A modification are associated with HCC progression by affecting various aspects of lncRNA metabolism, including structure, maturation, stability, splicing, export, translation and decay [6, 7]. For example, study of Tian Lan et al*.* proposed a complex KIAA1429-GATA3 regulatory model based on m^6^A modification and provides insights into the epi-transcriptomic dysregulation in HCC progression [8]. And Xueliang Zuo et al. found that m^6^A-mediated upregulation of LINC00958 increases lipogenesis and acts as a nanotherapeutic target in HCC [9].

LncRNAs is a kind of noncoding RNAs with the length of more than 200 nt, and without the ability to code proteins [10]. Specific patterns of lncRNA expression modulate cancer progression, including HCC [11]. It has been widely reported that dysregulated lncRNAs are involved in HCC cells metastasis, cell proliferation and cell apoptosis and so on [11]. For example, highly expressed lncRNA MCM3AP-AS1 promotes the growth of HCC by targeting miR-194-5p/FOXA1 axis. And lncTCF7 promotes self-renewal of human liver cancer stem cells through activation of Wnt signaling [12].

In this study, our data identified lncRNA glucosylceramidase beta pseudogene 1 (GBAP1) as the downstream target of METTL3, which positively regulates GBAP1 expression in an m^6^A-dependent manner. And upregulated GBAP1 is related to malignant clinicopathological features and poorer prognosis of HCC. Further experiments both in *vitro* and in *vivo* indicate that GBAP1 accelerates cell migration, invasion and growth by activating BMP/SMAD pathway under the mediation of miR-22-3p. Our findings reveal the essential regulatory role of METTL3/GBAP1/miR-22-3p/BMPR1A/SMAD signaling axis and highlight the great importance of m^6^A modification in modulating GBAP1 expression post-transcriptionally in HCC.

## Materials and methods

### Tissue samples

HCC tissue samples and adjacent non-tumor tissue samples, which were histopathologically confirmed, were collected from 85 HCC patients underwent surgery in the First Affiliated Hospital of Xi’an Jiaotong University from June 2015 to December 2017. All of the patients did not receive chemotherapy or radiotherapy before surgery. All of the samples were stored at − 80 °C. Our study got approval from the Ethics Committees of the First Affiliated Hospital of Xi’an Jiaotong University, and informed consent was obtained from all of the patients.

### Cell culture

The human normal liver cell line (LO2), five cell lines (HepG2, Huh7, Hep3B, MHCC97H and SMMC-7721) and human embryonic kidney (HEK) 293T cells were obtained from the Cell Bank of the Chinese Academy of Sciences (Shanghai, China). All of the cells were maintained in incubator (37 °C, 5% CO_2_), and cultured in DMEM (Gibco, Grand Island, NY, USA) supplemented with 10% FBS (Gibco, Grand Island, NY, USA) and 1% penicillin–streptomycin (Invitrogen, CA, USA).

### Plasmids, mutation and cell transfection

GBAP1 overexpressing plasmid, BMPR1A overexpressing plasmid, METTL3 overexpressing plasmid and empty pcDNA3.1 vector were obtained from RiboBio (Guangzhou, China). The small hairpin RNAs (shRNAs) targeting GBAP1 (shGBAP1#1 and shGBAP1#2) were obtained from Genechem (Shanghai, China). The small hairpin RNAs (shRNAs) targeting IGF2BP2 (shIGF2BP2#1 and shIGF2BP2#2), BMPR1A (shBMPR1A#1 and shBMPR1A#2) and METTL3 (shMETTL3#1 and shMETTL3#2) were obtained from Sigma-Aldrich. Full-length of GBAP1 (wild type, wt) or m^6^A mutant (mutant 1, mutant 2, mutant 3, mutant 4) GBAP1 sequences were cloned into psiCHECKTM-2 Vector (Promega, USA). All sequences were verified by DNA Sanger sequencing. The transfected cells were treated with 5 μg/ml puromycin and 800 μg/ml neomycin to select knockdown and overexpressed single clone cells. MiR-22-3p mimics, miR-22-3p inhibitors, along with NC mimics and NC inhibitor were obtained from Obio Technology (Shanghai, China). The transfections were performed by using Lipofectamine 3000 reagent (Invitrogen, CA, USA) according to the manufacturer’s instructions.

### Quantitative real-time PCR (RT-qPCR)

Isolation of total RNA was conducted by using TRIzol reagent (Thermo Fisher Scientific) and was reverse-transcribed into cDNA by a Reverse Transcription Kit (Invitrogen, CA, USA). Real-time PCR analysis was performed using SYBR Green Premix PCR Master Mix (Roche, Mannheim, Germany) under ABI HT9600 (Applied Biosystems, Foster City, CA, USA). The relative expression level was normalized to GAPDH or U6 and was calculated by 2^−ΔΔCt^ methods. Primers for GBAP1, IGFBP2, BMPR1A, METTL3, GAPDH, miR-22-3p and U6 were purchased FulenGen (Guangzhou, China).

### Western blot

Total protein was isolated from cells with RIPA buffer (Beyotime, Hangzhou, China). Protein was separated by 10% SDS-PAGE gels, then transferred to PVDF membranes (Millipore, Billerica, MA, USA). After being blocked by 5% nonfat milk for 2 h, antibodies for METTL3 (1:1000, #ab195352, Abcam, USA), IGF2BP2 (1:1000, # ab129071, Abcam, USA), BMPR1A (1:1000, # ab174815, Abcam, USA), Phospho-Smad1/5 (Ser463/465) (41D10) (1:1000, #9516, Cell Signaling Technology, Inc., MA, USA), SMAD1 (1:1000, # ab63356, Abcam, USA), and β-actin (1:1000, # ab8226, Abcam, USA) were used to incubate membranes at room temperature overnight. Then, the membranes were incubated by the HRP-conjugated secondary antibodies. The blots were detected using enhanced chemiluminescence reagent (Millipore, Billerica, MA, USA).

### Transwell migration and invasion assays

For Transwell migration and invasion assays, the cells (8 × 10^4^) were seed into the 8-μm-pore Transwell inserts (Corning-Costar, Cambridge, MA) containing 200 μL serum-free medium. The lower chambers were added with 800 μL complete culture medium. For detection of invasion ability, Transwell chambers were pre-coated with 15 μL Matrigel (BD Biosciences, Bedford, MA). After incubation for 24–48 h, cells passed through the membrance were stained with crystal violet (0.1%) and counted.

### Wound healing assay

Transfected cells were seeded into 6-well plates to form cell monolayers. When cell confluency reached to 85%, a 200 μL tip was used to scratch the cell layers. After being gently washed, cells were cultured for 48 h. The wound was visualized and images were taken at 0 and 48 h with the inverted microscope (Nikon, Tokyo, Japan).

### MTT assay, EdU assay

For MTT assay, transfected cells were planted into 96-well plates (2000 cells/well). Then at 0, 24, 48, and 72 h after seeding, MTT (10 μL/well, Sigma, USA) was added to each well and incubated for 4 h at 37 °C. Then, DMSO (100 μL/well, Sigma, °C USA) was used to dissolve the crystals. Absorbance was measured at 490 nm by a microplate reader (Bio-Rad, Richmond, CA). For EdU assay, Cell-Light™ EdU Apollo®567 In Vitro Imaging Kit (RiboBio Co., Ltd. Guangzhou, China) was used. Briefly, transfected HCC cells (1 × 10^⁠5^) were cultured in 96-well plates. Cells were incubated with EdU labelling medium at moderate concentration for 2 h. Then, the cells were fixed with 4% paraformaldehyde, glycine and 0.5% TritonX-100 in PBS. Next, cells were stained with 100 μL Apollo dye solution for 30 min at room temperature. The cells were subsequently stained using Hoechst and incubated for 30 min. The photos were taken on a microscope. The percentage of EdU positive cells was calculated using ImageJ software.

### Luciferase reporter assay

QuikChange® Site‑Directed Mutagenesis kit (STRATAGENE, Shanghai, China) was used to generate the mutants of GBAP1-3'UTR and BMPR1A-3'UTR.The pGL3 luciferase reporter vectors were used to construct pGL3-GBAP1-wt/pGL3-BMPR1A-wt plasmid and pGL3-GBAP1-mut/pGL3-BMPR1A-mut plasmid. Then, pGL3-GBAP1-wt/pGL3-BMPR1A-wt or pGL3-GBAP1-mut/pGL3-BMPR1A-mut was co-transfected with miR-22-3p mimics or anti-miR-22-3p plasmid into HEK293T cells. After 48 h, luciferase activity detection was conducted with the dual luciferase reporter assay system (Promega Corporation, Fitchburg, WI, USA).

### RNA-seq analysis

Hep3B-Vector and Hep3B-pcDNA/METTL3 subclones were seeded into six-well plates in three biological replicates. Total RNA was isolated from the subclones using TRIzol (Invitrogen) and treated with deoxyribonuclease (Qiagen). Library preparation and sequencing using the NovaSeq 6000 platform (Illumina) were performed. The FASTQ files were subjected to quality check and analyzed by Genialis Inc. (https://www.genialis.com). Differential expression results with a false discovery rate of < 0.05 and mRNA fold change of > 2 was used as a cutoff for further downstream analysis.

### MeRIP assay

The commercial Magna MeRIP™ m^6^A Kit (Millipore, USA) was used perform MeRIP assays according to the manufacturer’s protocol. Total RNA was fragmented in fragmentation buffer. Then, the m^6^A antibody and Magna ChIP Protein A/G Magnetic Beads were added to above buffer and rotated at room temperature for 30 min. Beads that captured the m^6^A antibody and MeRIP reaction mixture that contained fragmented RNA, RNase buffer and IP buffer Antibody were rotated for 2 h at 4 °C. The needed RNA was obtained from beads using elution buffer at 4 °C with continuous shaking for 1 h. RNA was extracted and purified. Immunoprecipitated RNAs were analyzed using qRT-PCR.

### RNA immunoprecipitation (RIP)

RIP assay was performed according to the manufacturer's instructions of EZ-Magna-RIP kit (Millipore, Billerica, USA). Cells were lysed by lysis buffer. Then, cell lysates were pre-cleaned with recombinant protein A/G agarose (Thermo Fisher Scientific) to minimize non-specific binding for 30 min at 4 °C. One to ten percent of the samples were used as input. Equal amount of cell lysates was incubated with antibody against IgG or METTL3 overnight at 4 °C. IgG was used as a negative control. The RNA–protein/antibody complexes were captured by incubation with recombinant protein A/G agarose (Thermo Fisher Scientific). RNA was eluted from the precipitated complex and transcribed into cDNA. RT-qPCR assay was performed to detect binding of RNA to proteins or antibody.

### RNA half-life assay

The HCC subclones were treated with treated with Actinomycin D (5 μg/mL, Merck-Millipore, Darmstadt, Germany) to block the synthesis of new RNA. Cells were then harvested to extract with total RNA at 0 h, 2 h, 4 h, 6 h, 8 h after Actinomycin D addition for RT-qPCR analysis.

### Animal experiments

In vivo tumor growth assay, 1 × 10^7^ Hep3B-GBAP1 or MHCC97H-shGBAP1 subclones and the corresponding control cells were transplanted into the body of 6-week-old BALB/c nude mice (Slac Laboratory Animal Center, Shanghai, China) via subcutaneous injection. Tumor size was measured every 3 days. Twenty-one days later, tumors were removed from mice in different groups. Tumor weight was calculated after dissection. Permission of conducting animal study was obtained from the Research Ethics Committee of Xi’an Jiaotong University. In vivo lung metastasis model, 1 × 10^6^ cells were intravenously injected into the lateral tail vein of nude mice (n = *5* mice/group). After 5 weeks, the mice were sacrificed. Thereafter, to analyze the presence of metastatic nodules, the lungs were fixed, photographed, preserved, and stained with hematoxylin and eosin.

### Immunohistochemistry and hematoxylin–eosin staining

For immunohistochemistry, xenograft tumors were fixed with paraformaldehyde and embedded in paraffin. The samples were sectioned and mounted on slides. The sectioned slides were incubated by antibody against Ki-67 (1:500, ab15580, Abcam, USA) at 4 °C overnight. Then the sections were incubated with biotinylated secondary antibody bound to a horseradishperoxidase complex. The antibody was visualized by adding 3,3-diaminobenzidine, and the sections were counterstained with hematoxylin. For Hematoxylin–Eosin staining, lung tissues in mice were collected and washed with PBS, followed by fixed in 10% neutral formalin solution (Sigma-Aldrich) and embedded in paraffin (Sigma-Aldrich). Tissues were sliced, after which were stained by Hematoxylin–Eosin (HE) (Sigma-Aldrich).

### Statistical analysis

Graphpad Prism 6.0 (San Diago, CA, USA) and SPSS 20.0 software (SPSS, Inc., Chicago, IL, USA) were applied to analyze the data. All of the data are presented as mean ± S.D. Statistical methods in this study included Student’s t test, one-way ANOVA, Chi-square test, Kaplan–Meier method, log-rank test and Pearson's correlation coefficient analysis and so on. Difference with *P* < 0.05 was considered to be statistically significant.

## Results

### Upregulated METTL3 increases GBAP1 expression in HCC

**Fig. 1:**
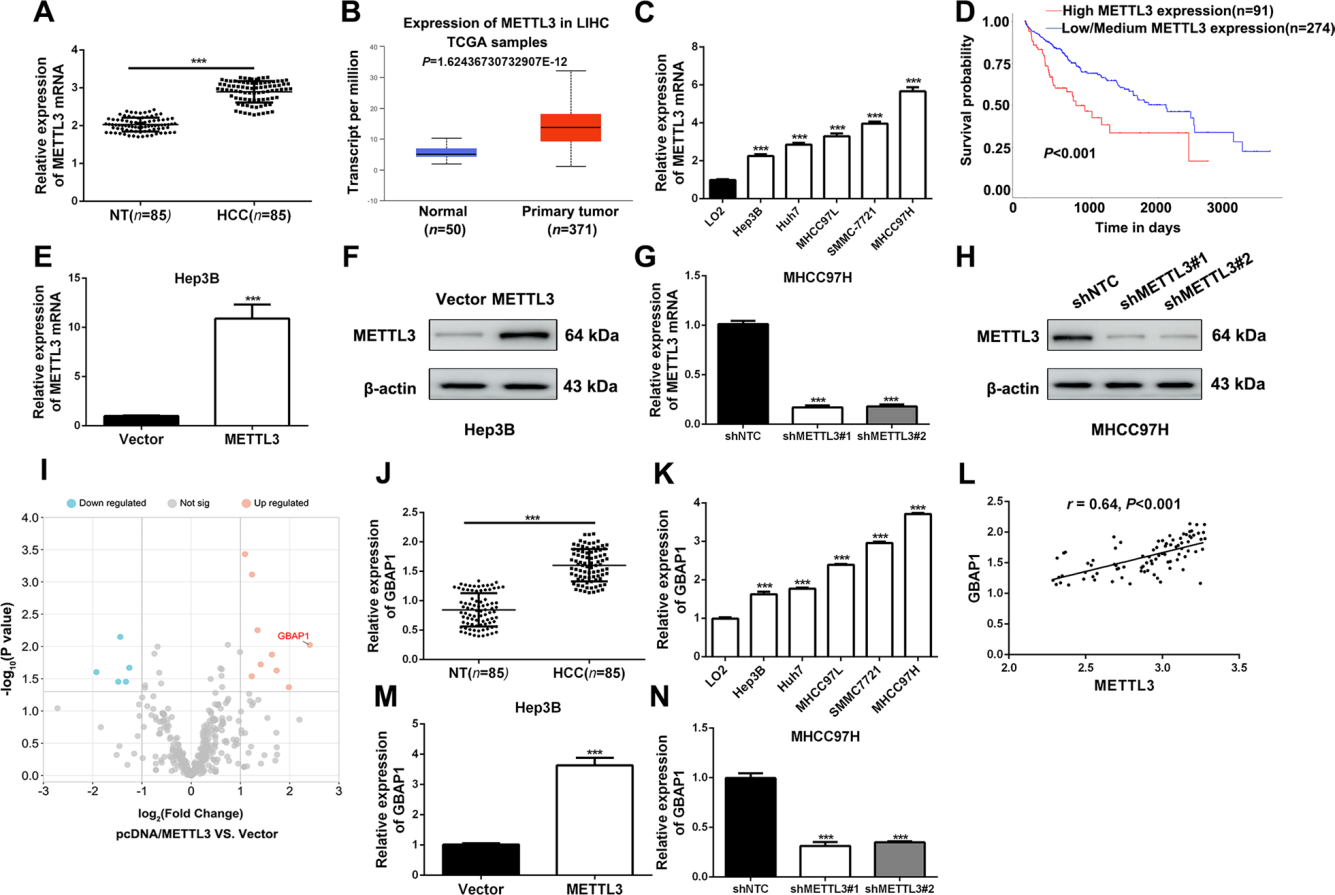
Upregulated METTL3 increases GBAP1 expression in HCC. **A** RT-qPCR analysis was applied to explore METTL3 expression in HCC tissues (*n* = 85) and adjacent non-tumor tissues *(n* = 85) (mean ± SD; *n* = 3). ***P < 0.001 (Student’s t test). **B** Data from TCGA showed that METTL3 expression was significantly increased in HCC tissues (*n* = 371) compared to normal tissues (*n* = 50) (https://ualcan.path.uab.edu/index.html) (Student’s t test). **C** RT-qPCR analysis was applied to explore METTL3 expression in HCC cell lines and normal hepatic cell LO2. ***P < 0.001 versus LO2 (two-way ANOVA). **D** Data from UALCAN platform showed that HCC patients with higher METTL3 expression had worse prognosis than that with lower METTL3 (https://ualcan.path.uab.edu). **E** and **F** RT-qPCR analysis and Western blot were applied to verify the overexpressing efficiency of pcDNA/METTL3 in Hep3B cells. ***P < 0.001 (Student’s t test). **G** and **H** RT-qPCR analysis and Western blot were applied to verify the knockdown efficiency of METTL3 shRNA#1 and shRNA#2 in MHCC97H cells. ***P < 0.001 versus shNTC (two-way ANOVA). **I** RNA-seq was conducted in METTL3 overexpressing and the control sunclones of Hep3B. Scatter diagram was applied to show the differentially expressed genes which were regulated by METTL3 in Hep3B cells. **J** RT-qPCR analysis was applied to explore GBAP1 expression in HCC tissues (*n* = 85) and adjacent non-tumor tissues (*n* = 85). ***P < 0.001 (Student’s t test). **K** RT-qPCR analysis was applied to explore GBAP1 expression in HCC cell lines and normal hepatic cell LO2. ***P < 0.001 versus LO2 (two-way ANOVA). **L** Pearson correlation analysis showed that there existed a positive correlation between METTL3 and GBAP1 in HCC tissues (*n* = 85). **M** and **N** RT-qPCR analysis showed that GBAP1 was significantly increased by pcDNA/METTL3 in Hep3B cells, while GBAP1 was significantly decreased by METTL3 shRNAs. ***P < 0.001 (Student’s t test). ***P < 0.001 versus shNTC (two-way ANOVA)

It has been reported that m^6^A modification influences the progression of HCC, and METTL3 plays crucial roles [13]. Here, METTL3 mRNA expression was found to be significantly increased in HCC tissues of our cohort of 85 patients by RT-qPCR analysis (Fig. 1A), which was consistent with the data analysis from TCGA (Fig. 1B). In addition, expression was significantly increased in HCC cell lines (Fig. 1C and Additional file 1: Fig. S1). Clinically, HCC patients with higher METTL3 expression had worse prognosis (Fig. 1D). Then, the overexpressed or knockdown METTL3 subclones of Hep3B or MHCC97H cells were constructed and the efficiency was verified by RT-qPCR analysis and western blot (Fig. 1E–H).

To explore how METTL3 mediated HCC progression, we applied RNA sequencing (RNA-seq) in Hep3B sunclones overexpressing METTL3 or control vector. LncRNA GBAP1 appeared to be the top hit because of the most remarkable fold change in the upregulated targeted-gene group (Fig. 1I and Additional file 2), suggesting GBAP1 might be positively regulated by METTL3 in HCC. In our cohort of 85 patients, the expressions of GBAP1 in HCC tissues and adjacent non-tumor tissues were investigated by RT-qPCR. Data indicated that GBAP1 was significantly higher in HCC tissues than that in non-tumor tissues (Fig. 1J). Besides, RT-qPCR results in HCC cell lines revealed that GBAP1 expression in all of the five HCC cell lines (Hep3B, Huh7, MHCC97L, SMMC7721 and MHCC97H) were dramatically higher than that in human normal liver cell line (LO2) (Fig. 1K). As expected, METTL3 expression was positively correlated with GBAP1 in HCC tissues (Fig. 1L), which was also demonstrated by the consistent expression ranks in a series of HCC cell lines for these two genes (Fig. 1C, K). Additionally, METTL3 overexpressing remarkably increased GBAP1 expression of Hep3B cells and silenced METTL3 significantly decreased GBAP1 expression of MHCC97H cells (Fig. 1M, N). Taken together, the above findings demonstrated that upregulated METTL3 increases GBAP1 expression in HCC.

### METTL3 induces GBAP1 expression in an m^6^A-dependent manner in HCC

**Fig. 2:**
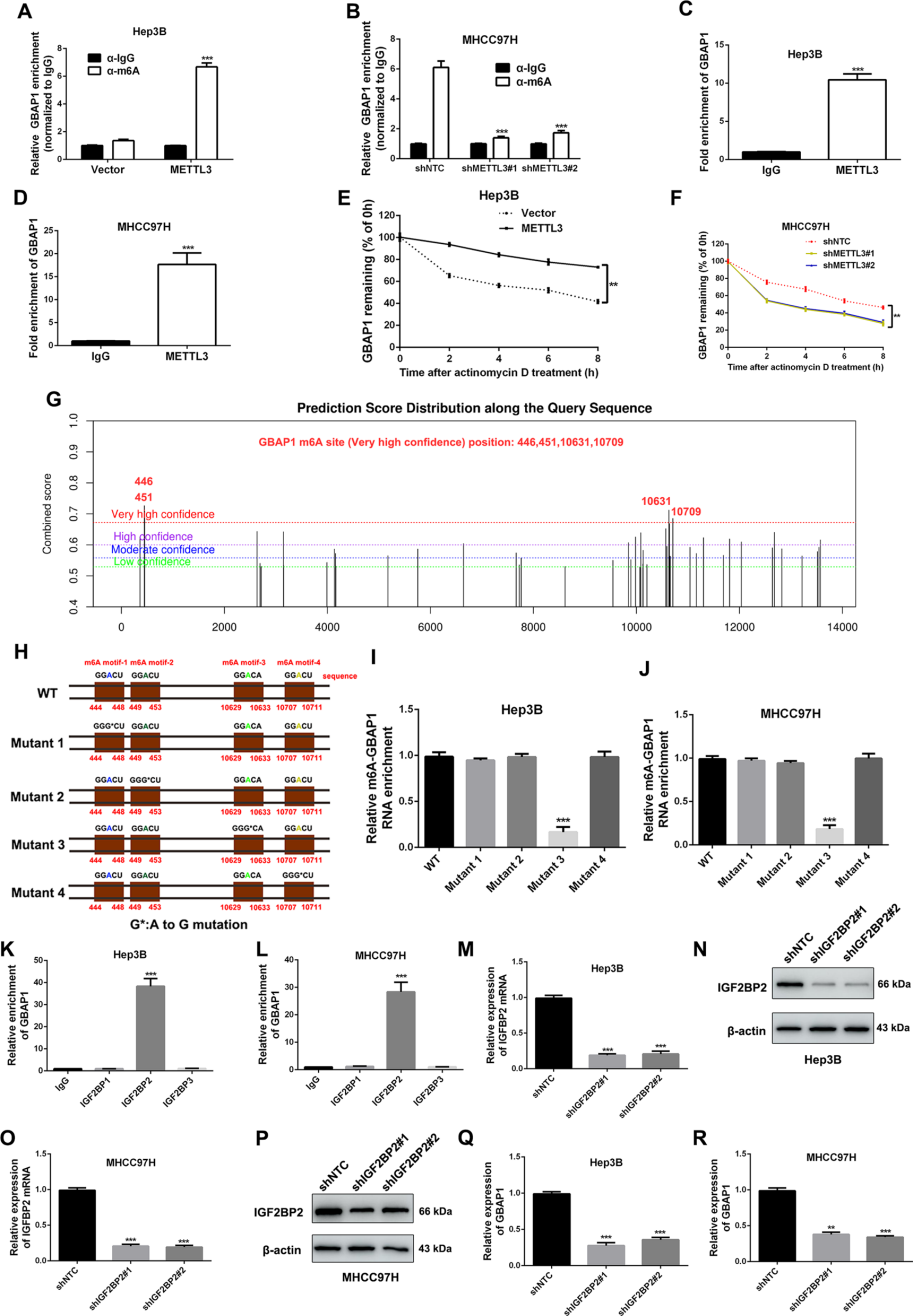
METTL3 induces GBAP1 expression in an m^6^A-dependent manner in HCC. **A** and **B** MeRIP-qPCR was conducted in METTL3 overexpressing subclones of Hep3B cells and METTL3 knockdown subclones of MHCC97H cells by using antibody against m^6^A or IgG. Primers of GBAP1 genes were used for qPCR. ***P < 0.001 (Student’s t test). ***P < 0.001 versus shNTC (two-way ANOVA). **C** and **D** RIP assay was conducted in Hep3B cells and MHCC97H cells. Primers of GBAP1 genes were used for qPCR. ***P < 0.001 (Student’s t test). **E** and **F** Hep3B or MHCC97H was treated with actinomycin D (10 μg/mL), an inhibitor of RNA polymerase elongation. RT-qPCR analysis was conducted to test GBAP1 expression level at 0, 2, 4, 6, 8 h after the treatment. **P < 0.01 (two-way ANOVA with Sidak’s t test). **G** The potential m^6^A site positions of GBAP1 were predicted by SRAMP platform (http://www.cuilab.cn/sramp). Four potential m^6^A sites with very high confidence (446A, 451A, 10631A and 10709A) were identified. **H** The plasmids with the A-to-G mutation at the 446, 451, 10,631 and 10,709 base of GBAP1-WT (wild type) were constructed. **I** and **J** MeRIP-qPCR was conducted in different GBAP1-mutated GBAP1-WT subclones of Hep3B and MHCC97H cells. Primers of GBAP1 genes were used for qPCR. ***P < 0.001 versus WT (two-way ANOVA). **K** and **L** RIP assay was conducted in Hep3B cells and MHCC97H cells. Primers of GBAP1 genes were used for qPCR. ***P < 0.001 versus IgG (two-way ANOVA). **M**–**P** IGF2BP2 knockdown subclones of Hep3B and MHCC97H cells were constructed. RT-qPCR and western blot were applied to test the efficiency. ***P < 0.001 versus shNTC (two-way ANOVA). **Q** and **R** RT-qPCR analysis was used to test the effect of IGF2BP2 knockdown on GBAP1 expression in Hep3B or MHCC97H cells. ***P < 0.001 versus shNTC (two-way ANOVA)

As an m^6^A writer, METTL3 is a critical mRNA methyltransferases [14]. We attempted to explore whether METTL3 increased GBAP1 expression through METTL3-mediated m^6^A modification in HCC cells. MeRIP-qPCR showed that the m^6^A level of GBAP1 in Hep3B cells was increased by pcDNA/METTL3 (Fig. 2A), while decreased by METTL3 shRNAs (Fig. 2B). Immunoprecipitation assay using antibody against METTL3 showed that GBAP1 was enriched by METTL3 antibody both in Hep3B and MHCC97H cells (Fig. 2C, D). Furthermore, Hep3B or MHCC97H was treated with actinomycin D (10 μg/mL), an inhibitor of RNA polymerase elongation. In the time courses, we found that decay rate of GBAP1 in Hep3B was slower when METTL3 was overexpressed (Fig. 2E), while the speed in MHCC97H was faster when METTL3 was silenced (Fig. 2F). By using the SRAMP algorithm (http://www.cuilab.cn/sramp), we identified four potential m^6^A sites with very high confidence (446A, 451A, 10631Aand 10709A) of ARHGAP5-AS1 RNA (Fig. 2G). Then the plasmids with the A-to-G mutation at the 446, 451, 10,631 and 10,709 base sites of WT were constructed (Fig. 2H). Subsequently, MeRIP-qPCR indicated that m^6^A levels of GBAP1 were significantly reduced both in Hep3B and MHCC97H cells with ectopic expression of the GBAP1 mutant 3 compared to the cells with ectopic WT GBAP1 expression (Fig. 2I, J). The m^6^A readers IGF2BPs (IGF2BP1/2/3) have been reported to paly critical roles in mediating m^6^A modification. Thus, we attempted to identify whether IGF2BPs (IGF2BP1/2/3) acted as m^6^A readers in METTL3-mdiated m^6^A modification of GBAP1. The RIP-qPCR analysis showed that IGF2BP2 was the reader protein with the highest binding affinity with lncRNA GBAP1 both in Hep3B and MHCC97H cells (Fig. 2K, L). Then the IGF2BP2 knockdown subclones of Hep3B and MHCC97H were constructed by IGF2BP2 shRNA#1 and shRNA#2, which were verified by both RT-qPCR and western blot (Fig. 2M–P). As expected, GBAP1 expressions were dramatically decreased by IGF2BP2 shRNAs (Fig. 2Q, R). Collectively, the above findings demonstrate that METTL3 induces GBAP1 expression in an m^6^A-dependent manner under the mediation of IGF2BP2 in HCC cells.

### Overexpressed GBAP1 is associated with poor prognosis of HCC patients

**Fig. 3:**
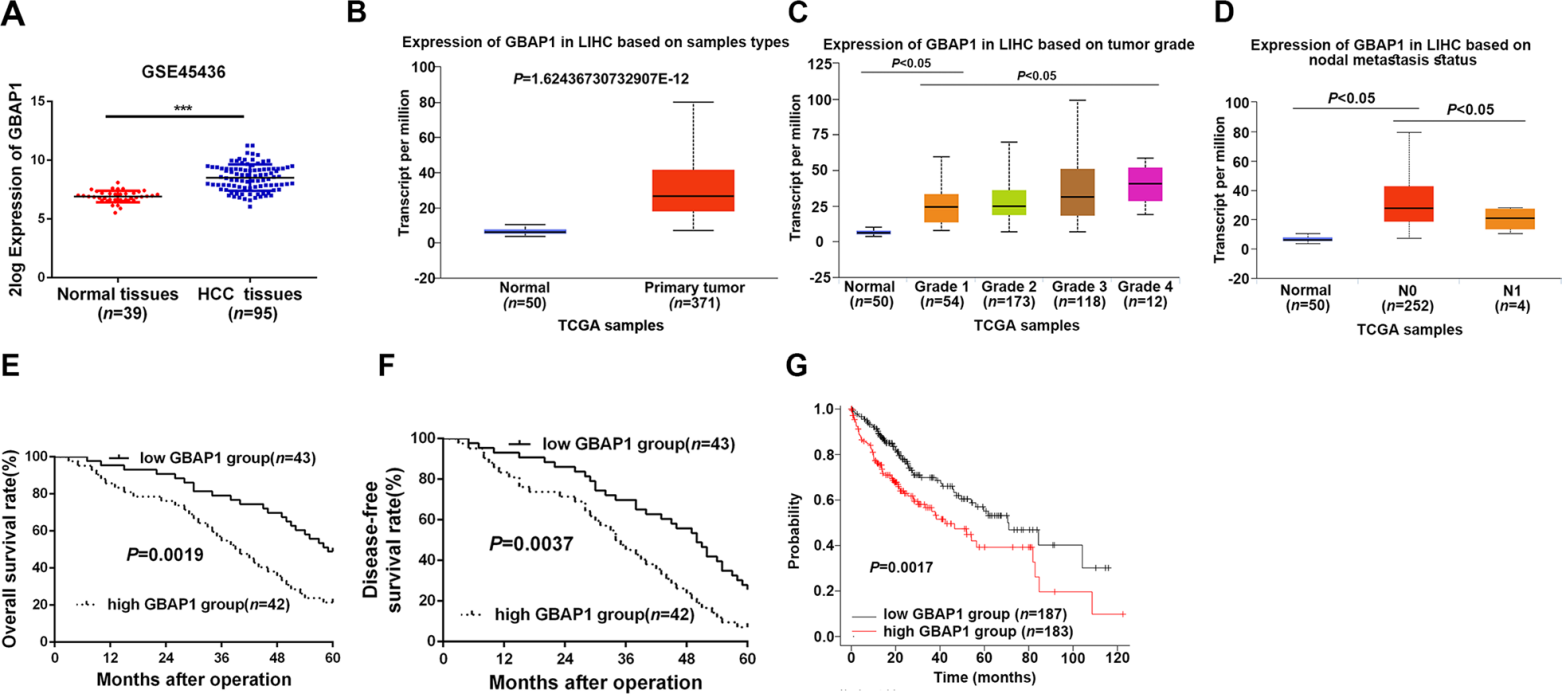
Overexpressed GBAP1 is associated with poor prognosis of HCC patients. **A** and **B** Data from GSE45436 and TCGA (https://ualcan.path.uab.edu/index.html) showed that GBAP1 was significantly increased in HCC. **C** and **D** GBAP1 expressions in HCC based on tumor grade or nodal metastasis was analyzed by TCGA platform (https://ualcan.path.uab.edu/index.html). **E** and **F** Kaplan–meier curves were established to explore the effects of GBAP1 expression on HCC patients 5-year overall survival and disease-free survival. **G** Public database platform Kaplan–Meier Plotter (https://kmplot.com/analysis) was applied to analyze the effects of GBAP1 expression on HCC patient prognosis

As shown in Fig. 1J and K, GBAP1 expression was significantly overexpressed in HCC tissues, which was consistent with the data form GEO dataset GSE45436 (Fig. 3A), TCGA data (Fig. 3B) and GEO dataset GSE84005 (Additional file 1: Fig. S2A). In addition, GBAP1 expression was gradually increased with the progression of tumor grade and nodal metastasis (Fig. 3C, D). In order to explore the clinical significance of upregulated GBAP1 in HCC, we categorized the 85 patients into two subgroups (low/high GBAP1 group) based on the median expression of GBAP1 in HCC tissues. As showed in Table 1, results of Chi-square test revealed that GBAP1 was significantly associated with tumor size (*P* = 0.022, Table 1), venous infiltration (*P* = 0.046, Table 1), and TNM stage (*P* = 0.041, Table 1). Additionally, higher GBAP1 expression was notably related to poorer 5-year overall survival (OS) (Fig. 3E) and disease-free survival (DFS) (Fig. 3F), which were consistent with the data from public platform Online Kaplan–Meier Plotter (Fig. 3G) and GEPIA (Additional file 1: Fig. S2B). Thus, the above findings demonstrate that GBAP1 is upregulated in HCC, and overexpressed GBAP1 is associated with poor prognosis of HCC patients.

**Table 1:** Correlation between GBAP1 expression and the clinicopathologic characteristics in HCC

Characteristics	Cases (*n* = 85)	Expression of GBAP1		*P*
		High (*n* = 42)	Low (*n* = 43)	
*Age (year)*				
< 50	27	12	15	0.532
≥ 50	58	30	28	
*Gender*				
Male	72	34	38	0.342
Female	13	8	5	
*HBV*				
Absent	12	9	3	0.056
Present	73	33	40	
*Serum AFP level (ng/mL)*				
< 400	21	14	7	0.068
≥ 400	64	28	36	
*Tumor size (cm)*				
< 5	39	14	25	0.022*
≥ 5	46	28	18	
*Number of tumor nodules*				
1	70	32	38	0.141
≥ 2	15	10	5	
*Cirrhosis*				
Absent	23	8	15	0.100
Present	62	34	28	
*Venous infiltration*				
Absent	61	26	35	0.046*
Present	24	16	8	
*Edmondson–Steiner grading*				
I + II	56	24	32	0.093
III + IV	29	18	11	
*TNM stage*				
I + II	63	27	36	0.041*
III + IV	22	15	7	

*P < 0.05

*HCC* hepatocellular carcinoma, *HBV* hepatitis B virus, *AFP* alpha-fetoprotein, *TNM* tumor-node-metastasis

### GBAP1 promotes HCC cells migration, invasion and growth in vitro

**Fig. 4:**
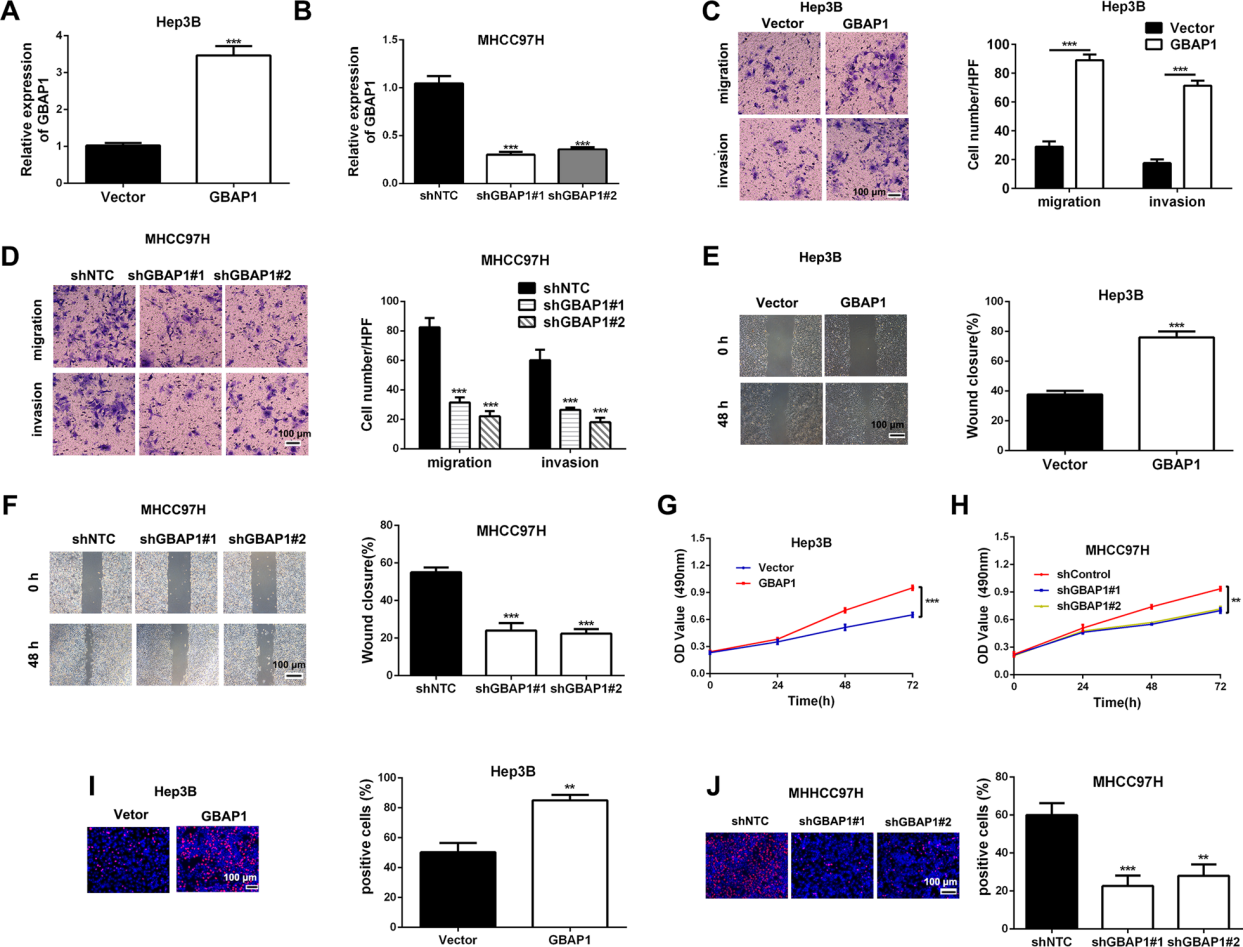
GBAP1 promotes HCC cells migration, invasion and growth in *vitro*. **A** pcDNA/GBAP1 was applied to overexpress GBAP1 expression of Hep3B cells. ***P < 0.001 (Student’s t test). **B** Lentiviral vectors encoding two different shRNAs (shGBAP1#1, shGBAP1#2) that targeted GBAP1 were stably transfected MHCC97H cells. RT-qPCR was used to test the knockdown efficiency. ***P < 0.001 versus shNTC (two-way ANOVA). **C** and **D** Transwell assays were used to measure the effects of GBAP1 overexpressing or knockdown on migration and invasion of Hep3B or MHCC97H cells. ***P < 0.001 (Student’s t test). ***P < 0.001 versus shNTC (two-way ANOVA). **E** and **F** Wound healing assay was applied to test the effect of GBAP1 overexpressing or knockdown on mobility of Hep3B or MHCC97H cells. ImageJ software was used to value the wound closure. ***P < 0.001 (Student’s t test). ***P < 0.001 versus shNTC (two-way ANOVA). **G** and **H** MTT assay was used to detect GBAP1 overexpressing or knockdown on viability of Hep3B or MHCC97H cells. **P < 0.01, ***P < 0.001 (two-way ANOVA with Sidak’s t test). **I** and **J** EdU assay was used to test the effects of GBAP1 overexpressing or knockdown on growth of Hep3B or MHCC97H cells. **P < 0.01 (Student’s t test). **P < 0.01, ***P < 0.001 versus shNTC (two-way ANOVA)

To explore the roles of GBAP1 in HCC cells metastasis, we respectively altered GBAP1 expression of Hep3B and MHCC97H with pcDNA/GBAP1 or GBAP1 shRNAs, and RT-qPCR was applied to confirm the overexpressing and knockdown efficiencies (Fig. 4A, B). Then, Transwell assays were conducted to detect the migration and invasion ability changes in Hep3B-GBAP1 and MHCC97H-shGBAP1 subclones compared to control groups. Both Transwell migration and invasion assays indicated that Hep3B cell number passing through the membrane or matrigel was notably increased by pcDNA/GBAP1 (Fig. 4C), while MHCC97H cell numbers was markedly decreased by GBAP1 shRNAs. (Fig. 4D). In addition, wound healing assay revealed that the mobility of Hep3B cells was significantly increased by pcDNA/GBAP1 (Fig. 4E), while the mobility of MHCC-97H cells was obviously suppressed by GBAP1 shRNAs (Fig. 4F). Epithelial-mesenchymal transition (EMT) is critical for migration and invasion of HCC cells [15]. Then, we attempted to explore whether GBAP1 had any effect on HCC cells EMT by testing EMT markers (E-cadherin, N-cadherin and Vimentin) change using western blot. The data indicated that epithelial cell marker (E-cadherin) was negatively regulated by GBAP1, while mesenchymal cell markers (N-cadherin and Vimentin) were positively regulated by GBAP1 (Additional file 1: Fig. S3). Next, we attempted to explore the effects of GBAP1 on cell growth of HCC cells. The results of MTT assay revealed that Hep3B cell viability was dramatically enhanced by pcDNA/GBAP1 (Fig. 4G), while cell viability of MHCC97H was significantly suppressed by GBAP1 shRNAs (Fig. 4H). Consistently, EdU assay showed that pcDNA/GBAP1 promoted proliferation of MHCC-97H cells (Fig. 4I), while GBAP1 shRNAs had the contrary effect on Hep3B cells (Fig. 4J). Besides, flow cytometry for detection of cell apoptosis indicated that pcDNA/GBAP1 inhibited apoptosis of Hep3B cells (Additional file 1: Fig. S4A), while shGBAP1 had the contrary effect on MHCC97H cells' apoptosis (Additional file 1: Fig. S4B). Taken together, we demonstrate that GBAP1 promotes cell growth of HCC cells.

### GBAP1 promotes HCC cells metastasis and cell growth in vivo

**Fig. 5:**
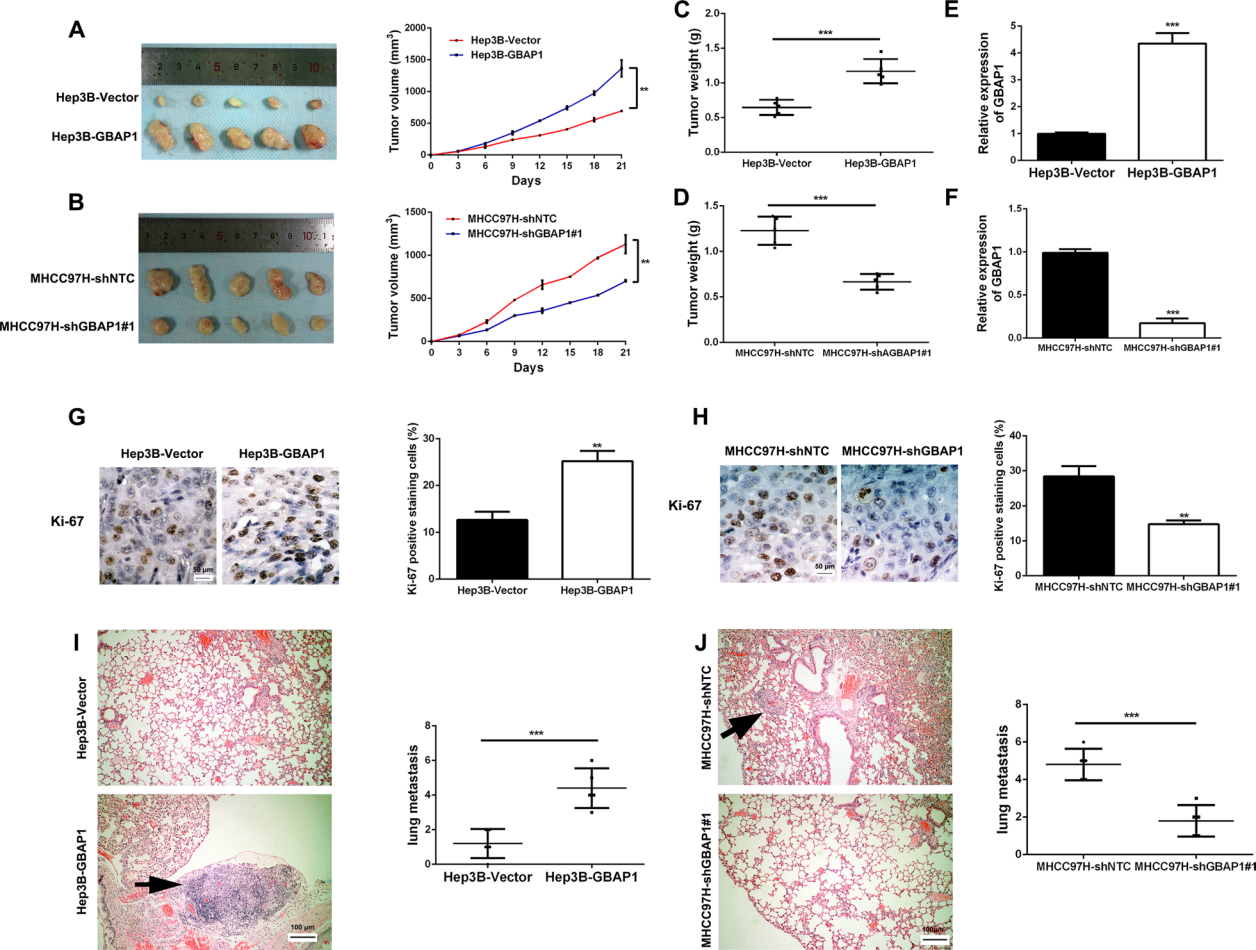
GBAP1 promotes HCC cells metastasis and cell growth in vivo. **A** and **B** Hep3B subclones stably overexpressing GBAP1 and or MHCC97H subclones stably expressing shRNA targeting GBAP1 or the control subclones were subcutaneously injected into the flanks of mice. Tumor size was measured every 3 days. Tumor nodules were removed from the flans after 3 weeks and growth curves were constructed. **P < 0.01 (two-way ANOVA with Sidak’s t test). **C** and **D** The weight of tumor nodules in different groups was measured. ***P < 0.001 (Student’s t test). **E** and **F** RT-qPCR analysis was applied to detect the expression of GBAP1 in mice tumor. ***P < 0.01 (Student’s t test). **G** and **H** Immunohistochemical staining was conducted with antibody against Ki-67 in mice tumor slides. ImageJ software was used to value the staining result. Magnification: × 400. **P < 0.01 (Student’s t test). **I** and **J** Hep3B subclones stably overexpressing GBAP1 and or MHCC97H subclones stably expressing shRNA targeting GBAP1 or the control subclones were used to constructed lung metastasis model via tail intravenous injection. After 5 weeks, hematoxylin–eosin staining was conducted in the lung tissue slides. ***P < 0.01 (Student’s t test)

In order to further explore whether GBAP1 promoted HCC cells metastasis and cell growth, we established the subcutaneous xenotransplanted tumor model and tail vein injection lung metastasis model in nude mice. As expected, the tumor growth in Hep3B-GBAP1 group was significantly promoted (Fig. 5A), while the MHCC97H-shGBAP1#1 group showed significantly slower tumor growth (Fig. 5B). Besides, the final tumor weight was dramatically increased in hep3B-GBAP1 group (Fig. 5C), while MHCC97H-shGBAP1#1 group had the decreased final tumor weight (Fig. 5D). Expression of GBAP1 in the tumor tissues was tested to validate that the differences in tumor growth and weight were leaded by the alteration of GBAP1 expression (Fig. 5E, F). Furthermore, immunohistochemistry for Ki-67 staining indicated that pcDNA/GBAP1 promoted tumor cells proliferation (Fig. 5G), while GBAP1 shRNA has the contrary effect (Fig. 5H). HE staining in lung tissues from tail vein injection lung metastasis model showed that pcDNA/GBAP1 promoted lung metastasis of Hep3B cells (Fig. 5I), while GBAP1 shRNA suppressed lung metastasis of MHCC97H cells (Fig. 5J). Taken together, the above findings demonstrate that GBAP1 promotes HCC cells metastasis and cell growth in *vivo.*

### GBAP1 acts as the molecular sponge of miR-22-3p in HCC cells

**Fig. 6:**
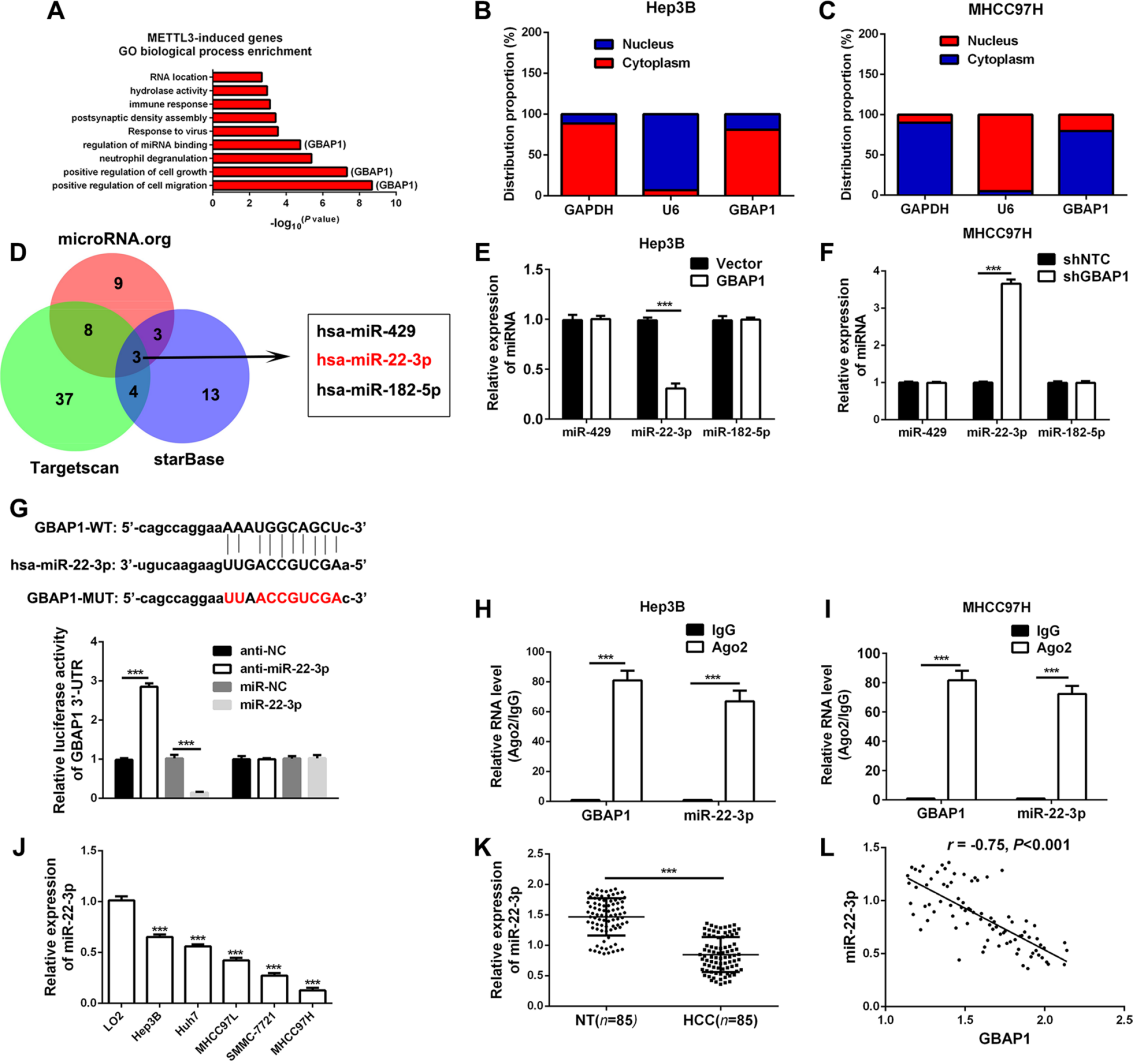
GBAP1 acts as the molecular sponge of miR-22-3p in HCC cells. **A** The upregulated genes by METTL3 based on RNA-seq data were subjected to Gene Ontology (GO) biological process analysis. The top 10 categories were shown. **B** and **C** Fractionation of nuclear and cytoplasmic RNA assay was applied to identify the subcellular localization of GBAP1 in Hep3B and MHCC97H cells. **D** Data overlap from three bioinformatics tools (Targetscan, microRNA.org and starBase) revealed that there were three potential miRNAs which could bind to GBAP1-3’UTR. And miR-22-3p was one of them. **E** and **F** RT-qPCR was applied to test the expressions of the potential miRNAs in Hep3B-GBAP1 subclones or MHCC97H-shGBAP1 subclone and the control subclones. ***P < 0.001 (Student’s t test). **G** The potential bind site between wild type of GBAP1 (GBAP1-WT) and miR-22-3p was identified by bioinformatics tools. And the mutant type of GBAP1 (GBAP1-MUT) was established. Double luciferase reporter gene assay revealed that miR-22-3p mimics repressed the fluorescence activity of GBAP1-3’UTR-WT, while miR-22-3p inhibitors enhanced the fluorescence activity of GBAP1-3’UTR-WT. The alteration of miR-22-3p expression had no effect on GBAP1-3’UTR-MUT. **H** and **I** RIP assay using antibody against Ago2 for pull-down showed that in Hep3B and MHCC97H cells both GBAP1 and miR-22-3p were enriched by Ago2 antibody. ***P < 0.001 (Student’s t test). **J** RT-qPCR was performed to analyze GBAP1 expression in HCC cell lines and normal hepatic cell LO2. ***P < 0.001 versus LO2 (two-way ANOVA). **K** The expression of miR-22-3p was explored by RT-qPCR in HCC tissues (*n* = 85) and non-tumor tissues (*n* = 85). ***P < 0.001 (Student’s t test). **L** Pearson correlation analysis showed that there existed a negative correlation between GBAP1 and miR-22-3p in HCC tissues (*n* = 85)

In order to explore the potential mechanism by which GBAP1 exerted its influences on metastasis and growth of HCC cells, the GO biological process enrichment analysis was conducted based on the data in the METTL3-related RNA-seq. The result revealed that GBAP1 was closely related to cells migration, growth and the miRNA binding (Fig. 6A). Thus, we hypothesized that GBAP1 played the roles in HCC cells by acting as ceRNA. In addition, the subcellular localization of GBAP1 in Hep3B and MHCC97H cells was conducted by fractionation of nuclear and cytoplasmic RNA assay. The results indicated that GBAP1 was mainly located in cytoplasm of HCC cells (Fig. 6B, C), which consistently suggested that GBAP1 probably played the roles in HCC cells by acting as ceRNA. Bioinformatics tools (Targetscan, microRNA.org and starBase) were employed to predict the miRNA candidate which potentially bond to GBAP1, and three miRNA candidate miR-429, miR-22-3p and miR-182-5p were obtained (Fig. 6D). Then, we attempted to test the three miRNAs expression changes in Hep3B-GBAP1 and MHCC97H-shGBAP1 subclones. And GBAP1 was overexpressed in Hep3B cells, only miR-22-3p expression was significantly decreased (Fig. 6E). Meanwhile, when GBAP1 was knocked down, only miR-22-3p was significantly increased (Fig. 6F). Data from the public databases showed that there existed potential binding site between wild type of GBAP1 (GBAP1-WT) and miR-22-3p, and the mutant type of GBAP1 (GBAP1-MUT) was established (Fig. 6G). Besides, we inhibited or overexpressed miR-22-3p expression of 293 T or Hep3B or MHCC97H cells by miR-22-3p inhibitors (anti-miR-22-3p) or miR-22-3p mimics (miR-22-3p), and the efficiencies were tested by RT-qPCR (Additional file 1: Fig. S5). Then, double luciferase reporter gene assay revealed that miR-22-3p was able to negatively regulate the luciferase activity of GBAP1-WT, but had no effect on GBAP1-MUT (Fig. 6G). Besides, RIP assay using antibody against Ago2 for pull-down showed that in Hep3B and MHCC97H cells both GBAP1 and miR-22-3p were enriched by Ago2 antibody (Fig. 6H, I). RT-qPCR showed that miR-22-3p was significantly downregulated both in HCC tissues and cell lines (Fig. 6J, K). And, there existed a negative correlation between GBAP1 and miR-22-3p (Fig. 6L). Besides, data from database Online Kaplan–Meier Plotter showed that patients with lower miR-22-3p expression had worse prognosis (Additional file 1: Fig. S6). Taken together, these data collectively demonstrate that GBAP1 acts as the molecular sponge of miR-22-3p in HCC cells.

### GBAP1 activates BMP/SMAD signaling under the mediation of miR-22-3p in HCC cells

**Fig. 7:**
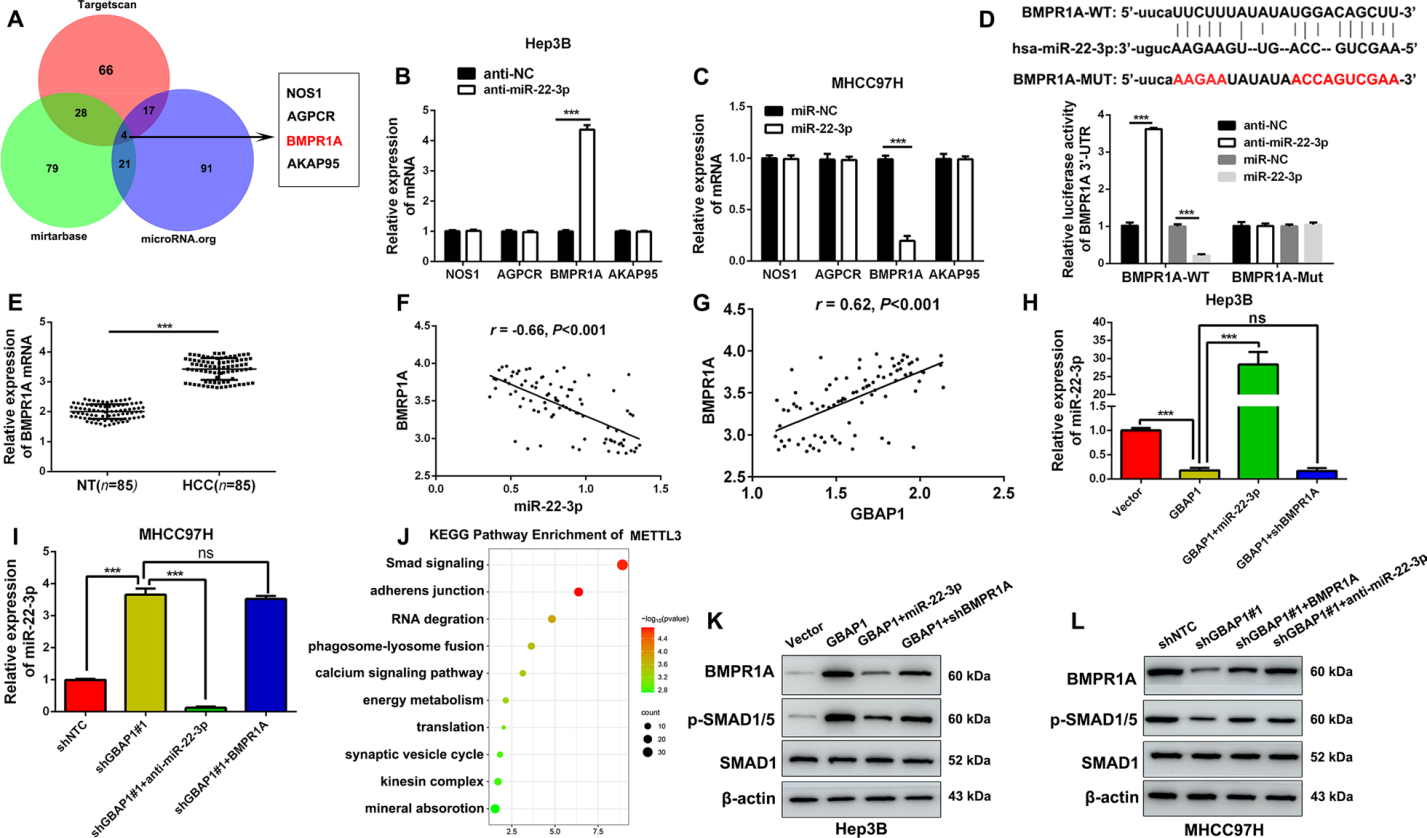
GBAP1 activates BMP/SMAD signaling under the mediation of miR-22-3p in HCC cells. **A** Three bioinformatics tools (TargetScan, mirtarbase and microRNA.org) were employed to predict the downstream target candidate of miR-22-3p, and four mRNA candidate NOS1, AGPCR, BMPR1A and AKAP95 were obtained.** B** and **C** The expression changes of four mRNAs in Hep3B-anti-miR-22-3p and MHCC97H-miR-22-3p subclones were tested by RT-qPCR. ***P < 0.001 (Student’s t test). **D** There existed potential binding site between wild type of BMPR1A (BMPR1A -WT) and miR-22-3p, and the mutant type of BMPR1A (BMPR1A-MUT) was established. Double luciferase reporter gene assay revealed that miR-22-3p was able to negatively regulate the luciferase activity of BMPR1A-WT, but had no effect on BMPR1A-MUT. ***P < 0.001 (Student’s t test). **E** RT-qPCR analysis was performed to analyze BMPR1A expression in HCC tissues (*n* = 85) and adjacent non-tumor tissues (*n* = 85). ***P < 0.001 (Student’s t test). **F** Pearson correlation analysis showed that there existed a negative correlation between BMPR1A and miR-22-3p in HCC tissues (*n* = 85). **G** Pearson correlation analysis showed that there existed a positive correlation between GBAP1 and BMPR1A in HCC tissues (*n* = 85). **H** and **I** miR-22-3p expression was negatively regulated by GBAP1 alone, while the effect of GBAP1 alone on miR-22-3p expression was reversed in GBAP1 + miR-22-3p group and shGBAP1#1 + anti-miR-22-3p group. **J** The KEGG pathway enrichment of GBAP1 based on RNA-seq data were analyzed. The top 10 categories were shown. **K** and **L** Western blot was applied to analyze the expression changes of BMPR1A, p-SMAD1/5 and SMAD1 in different co-transfection groups

In order to explore the potential mechanism by which miR-22-3p exerted its influences on metastasis and proliferation of HCC cells, bioinformatics tools (TargetScan, mirtarbase and microRNA.org) were employed to predict the downstream target candidate of miR-22-3p, and four mRNA candidate NOS1, AGPCR, BMPR1A and AKAP95 were obtained (Fig. 7A). Then, the four mRNAs expression changes in Hep3B-anti-miR-22-3p and MHCC97H-miR-22-3p subclones were tested by RT-qPCR. Data showed that when miR-22-3p was inhibited in hep3B cells, only BMPR1A expression was significantly increased (Fig. 7B). Meanwhile, when miR-22-3p was overexpressed, only BMPR1A was significantly decreased (Fig. 7C). Data from the public databases showed that there existed potential binding site between wild type of BMPR1A (BMPR1A-WT) and miR-22-3p, and the mutant type of BMPR1A (BMPR1A-MUT) was established (Fig. 7D). Then, double luciferase reporter gene assay revealed that miR-22-3p was able to negatively regulate the luciferase activity of BMPR1A-WT, but had no effect on BMPR1A-MUT (Fig. 7D). RT-qPCR showed that miR-22-3p was significantly downregulated both in HCC tissues and cell lines (Fig. 7E and Additional file 1: Fig. S7A), which was consistent with the data from GSE45436 and TCGA (Additional file 1: Fig. S7B, C). And, there existed a negative correlation between BMPR1A and miR-22-3p (Fig. 7F). Thus, these data collectively demonstrate that GBAP1 acts as the molecular sponge of miR-22-3p in HCC cells.

In addition, there existed a positive correlation between GBAP1 and BMPR1A in HCC (Fig. 7G). Consistently, the data of RT-qPCR and Western blot conducted in tumor nodules from subcutaneous xenotransplanted tumor models revealed that miR-22-3p was negatively regulated by GBAP1 (Additional file 1: Fig. S8A, B), while both mRNA and protein expression of BMPR1A were positively regulated by GBAP1 (Additional file 1: Fig. S8C–F). Additionally, as showed in Fig. 7H and I, miR-22-3p was negatively regulated by GBAP1 alone, while the effect of GBAP1 alone on miR-22-3p expression was reversed in GBAP1 + miR-22-3p group and shGBAP1#1 + anti-miR-22-3p group. And the KEGG Pathway Enrichment analysis of GBAP1 was performed, and Smad signaling pathway attracted our attention as the top one (Fig. 7J). Then, we assumed that BMP/SMAD pathway might involve in the regulation of HCC progression by GBAP1. BMPR1A, p-SMAD1/5 and SMAD1 were tested in different groups by Western blot. Results in Hep3B cells showed that when GBAP1 alone was overexpressed, the expression levels of BMPR1A and p-SMAD1/5 were increased, while the effects were abrogated when miR-22-3p mimics or BMPR1A shRNA existed simultaneously with pcDNA/GBAP1 (Fig. 7K and Additional file 1: Fig. S9A). And, results in MHCC97H cells showed that when GBAP1 alone was downregulated, the expression levels of BMPR1A and p-SMAD1/5 were decreased, while the effects were reversed when miR-22-3p inhibitors or pcDNA/BMPR1A existed simultaneously with GBAP1 shRNA. But there was no change in SMAD1 expression in the above subclones (Fig. 7L and Additional file 1: Fig. S9B). Taken together, we demonstrate that BMPR1A is the downstream target of miR-22-3p, and GBAP1 activates BMP/SMAD signaling under the mediation of miR-22-3p in HCC cells.

### GBAP1 exerts the effects on HCC cells through GBAP1/miR-22-3p/BMPR1A axis

**Fig. 8:**
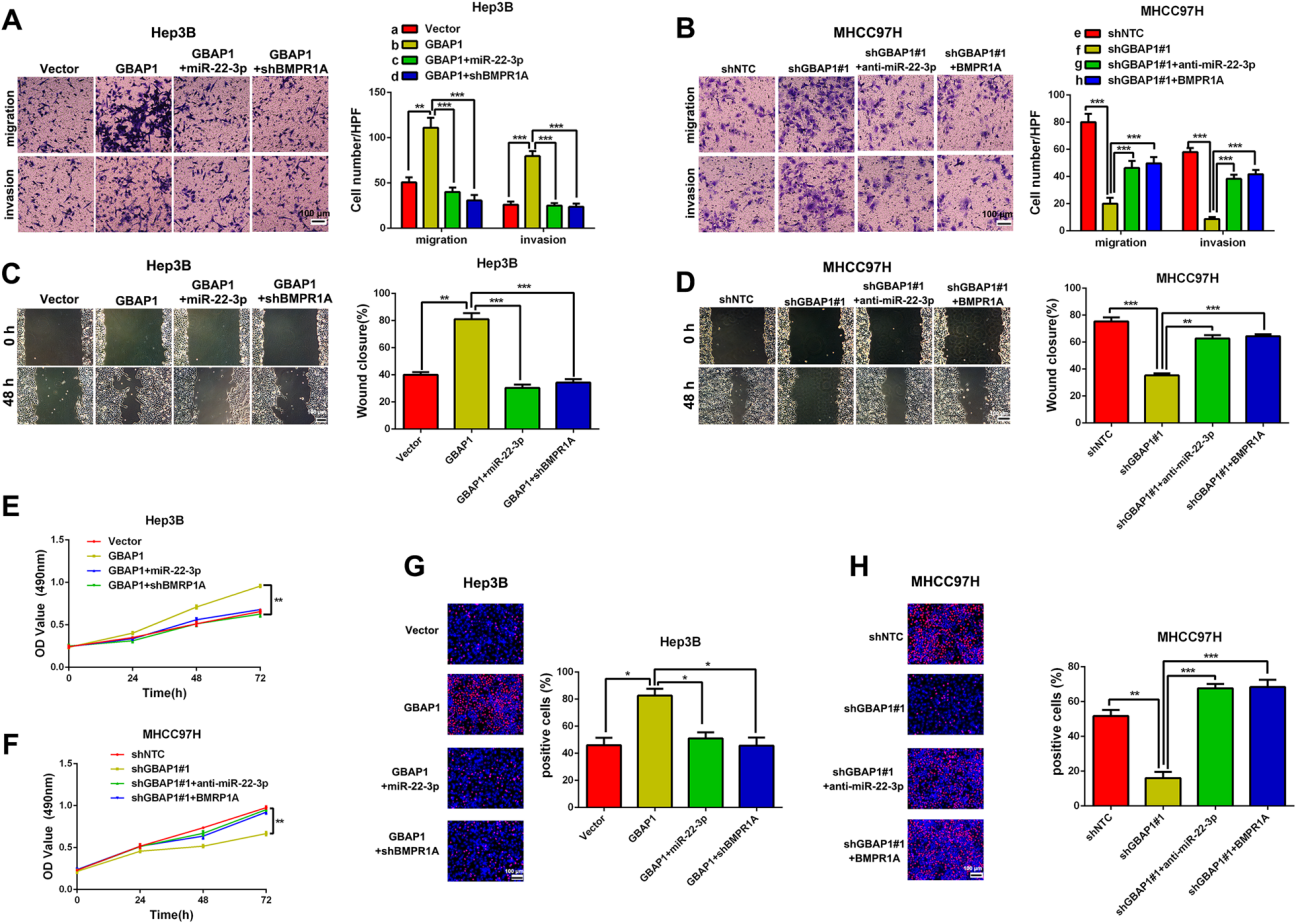
GBAP1 activates BMP/SMAD signaling under the mediation of miR-22-3p in HCC cells. **A** Transwell assay revealed that pcDNA/GBAP1 alone promoted migration and invasion of Hep3B cells, while miR-22-3p mimics or BMPR1A shRNA reversed the promotion effects of pcDNA/GBAP1 on migration and invasion of Hep3B cells. **P < 0.01 (Student’s t test). ***P < 0.001 (two-way ANOVA). **B** Transwell assay revealed that shGBAP1 alone inhibited migration and invasion of MHCC97H cells, while miR-22-3p inhibitors or pcDNA/BMPR1A reversed the inhibitory effects of shGBAP1 on migration and invasion of MHCC97H cells. ***P < 0.001 (Student’s t test). ***P < 0.001 (two-way ANOVA). **C** Wound healing assay revealed that pcDNA/GBAP1 alone promoted mobility of Hep3B cells, while miR-22-3p mimics or BMPR1A shRNA reversed the promotion effects of pcDNA/GBAP1 on Hep3B cells mobility. **P < 0.01 (Student’s t test). ***P < 0.001 (two-way ANOVA). **D** Wound healing assay revealed that shGBAP1 alone inhibited mobility of MHCC97H cells, while miR-22-3p inhibitors or pcDNA/BMPR1A reversed the inhibitory effects of shGBAP1 on MHCC97H cells mobility. ***P < 0.001 (Student’s t test). **P < 0.01, ***P < 0.001 (two-way ANOVA). **E** MTT assay revealed that pcDNA/GBAP1 alone promoted viability of Hep3B cells, while miR-22-3p mimics or BMPR1A shRNA reversed the promotion effects of pcDNA/GBAP1 on Hep3B cells viability. **P < 0.01 (two-way ANOVA with Sidak’s t test). **F** MTT assay revealed that shGBAP1 alone inhibited viability of MHCC97H cells, while miR-22-3p inhibitors or pcDNA/BMPR1A reversed the inhibitory effects of shGBAP1 on MHCC97H cells viability. **P < 0.01 (two-way ANOVA with Sidak’s t test). **G** EdU assay revealed that pcDNA/GBAP1 alone promoted Hep3B cells proliferation, while miR-22-3p mimics or BMPR1A shRNA reversed the promotion effects of pcDNA/GBAP1 on Hep3B cells proliferation. *P < 0.05 (Student’s t test). *P < 0.05 (two-way ANOVA). **H** EdU assay revealed that shGBAP1 alone inhibited MHCC97H cells proliferation, while miR-22-3p inhibitors or pcDNA/BMPR1A reversed the inhibitory effects of shGBAP1 on MHCC97H cells proliferation. **P < 0.01 (Student’s t test). ***P < 0.001 (two-way ANOVA). Magnification: 200 ×

Next, we attempted to investigate whether GBAP1 exerted its effects on migration, invasion and cell growth of HCC cells through GBAP1/miR-22-3p/BMPR1A axis in HCC cells. Rescue experiments of Transwell assays, Wound healing assay, MTT assay and EdU assay in Hep3B cells revealed that pcDNA/GBAP1 alone promoted migration, invasion, mobility, cell viability and proliferation of Hep3B cells, while the promotion effects were abrogated when miR-22-3p mimics or BMPR1A shRNA was co-transfected with pcDNA/GBAP1 (Fig. 8A, C, E, G). On the other hand, rescue experiments in MHCC97H cells showed that GBAP1 shRNA alone inhibited migration, invasion, mobility, cell viability and proliferation of MHCC97H cells, while the inhibitory effects were reversed when miR-22-3p inhibitors or pcDNA/BMPR1A was co-transfected with GBAP1 shRNA (Fig. 8B, D, F, H). Taken together, we demonstrate that GBAP1 exerts the effects on HCC cells migration, invasion and cell growth through GBAP1/miR-22-3p/BMPR1A axis.

## Discussion

HCC is one of the most challenging cancers worldwide, due to its strong abilities of migration, invasion and proliferation [16]. Accumulated evidences have demonstrated the critical roles of N6-methyladenosine (m^6^A) and lncRNAs in HCC [7]. As m^6^A methyltransferases, METTL3 interacts with METTL14 to form the METTL3/METTL14 methyltransferase complex, which convert A to m^6^A in RNAs in human cells [17]. Consistent with the previous researches [13], METTL3 was found to be upregulated in HCC and positively associated with the poor prognosis of HCC patients. After the genome-wide screening of lncRNAs in overexpressed METTL3 HCC subclones via RNA-seq, we identified GBAP1, which is also identified as a pseudogene and located at 1q22, as a novel potential m^6^A-modified lncRNA in HCC. Additionally, GBAP1 was positively regulated by METTL3 in an m^6^A-dependent manner in HCC, during which the m^6^A reader IGF2BP2 was found to be essential in mediating METTL3-induced m^6^A modification of GBAP1.

Originally, GBAP1 is identified to be associated with Parkinson's disease (PD). And, the further study reported that GBAP1 acts as a ceRNA by sponging miR-22-3p to regulate the expression of GBA gene, which encodes lysosomal glucocerebrosidase, the major predisposing factor for PD [18]. Recently, research in gastric cancer (GC) demonstrates that GBAP1 contributes to the development and progression of GC by sequestering the miR-212-3p from binding to GBA, which suggests that GBAP1 may also play roles in cancer progression [19]. In addition, Rong Chen et al. found that GBAP1 promotes HCC growth by inactivating the PI3K/AKT pathway [20]. In this study, GBAP1 was significantly highly expressed both in HCC tissues and cell lines. And, results indicated that GBAP1 was significantly related to tumor size, venous infiltration, TNM stage, and HCC patient prognosis. These findings suggest that GBAP1 is a valuable tumor biomarker for HCC, and it might act as an oncogene in HCC.

Then, gain-and loss-of-function analysis were performed to explore the biological functions of GBAP1 in HCC. Results of both in vitro and in vivo experiments manifested that downregulated GBAP1 inhibited HCC cells migration, invasion and growth, while overexpressed GBAP1 had the contrary effects. Then, we conclude that GBAP1 acts as a tumor promoter in HCC by accelerating cell migration, invasion and growth. In order to uncover the mechanism by which GBAP1 exerted its roles in HCC, GO biological process enrichment was performed. The data showed that GBAP1 might act as the sponges for miRNAs. More and more studies reveal the existence of a widespread interaction network involving ceRNAs, where lncRNAs act as the sponges for miRNAs to regulate the expression levels of specific mRNAs [21]. For example, lncRNA MCM3AP-AS1 exert its role in HCC by working as the sponge for miR-194-5p to regulated FOXA1 expression in HCC [12]. Thus, bioinformatics tools (Targetscan, microRNA.org and starBase), double luciferase reporter gene assay and RIP assay and so on, were applied to predict the potential miRNA that could bind to GBAP1. We found that as one of the three potential miRNAs, only miR-22-3p expression was negatively regulated by GBAP1. And there existed a negative correlation between miR-22-3p and GBAP1 expressions. Furthermore, in HCC cells, there existed direct interaction between GBAP1 and miR-22-3p in HCC cells. Thus, these findings suggest that GBAP1 acts as a sponge for miR-22-3p in HCC cells.

MiRNAs usually exert their influences on cancer cells via inhibition of transcription or promotion of degradation of the targeted mRNAs [22]. Then we attempted to explore the target by which miR-22-3p exerted its inhibitory influences on HCC cells. By bioinformatics tools (TargetScan, mirtarbase and microRNA.org), we searched for the most potential targets of miR-22-3p. Data from these databases collectively revealed that the BMPR1A was one of the potential targets of miR-22-3p. And, BMPR1A was the only one which was able to be negatively regulated by miR-22-3p. In addition, the KEGG Pathway Enrichment was performed, which indicated that the SMAD signaling might be involved in the regulation process of GBAP1 in HCC. It has been reported that the BMP/SMAD signaling plays a critical role in the regulation of HCC progression [23]. Activation of BMP signaling is mediated by ligand-induced heterotetrameric complex formation. BMPR1A is one of the receptors on target cell membranes, and play indispensable roles in transducing BMP signaling [24]. BMP/Smad signaling is a canonical pathway for BMP signaling, during which the phosphorylation of SMAD-1/5 acts as one of the activation markers of BMP signaling [25–27]. Here, we identified that BMPR1A was the downstream target of miR-22-3p in HCC cells. And BMPR1A expression and the phosphorylation level of SMAD-1/5 were found to be positively regulated by GBAP1 under the mediation of miR-22-3p. Furthermore, rescue experiments showed that artificially changing the expression of miR-22-3p and BMPR1A reversed the effects of GBAP1 on HCC cell metastasis and growth, as well as BMPR1A expression and the phosphorylation level of SMAD-1/5. Then, we conclude that GBAP1 promotes HCC progression by GBAP1/miR-22-3p/BMPR1A axis in HCC, and GBAP1 activates BMP/SMAD signaling under the mediation of miR-22-3p.

## Conclusions

This study identified an oncogenic lncRNA GBAP1 in HCC, which was induced by METTL3 in an m^6^A-dependent manner under the mediation of IGF2BP2. Clinically, overexpressed GBAP1 was closely associated with large tumor size, venous infiltration, advanced TNM stage and poorer prognosis. Functionally, GBAP1 acted as an oncogene by promoting migration, invasion and growth of HCC cells. In addition, we also elucidated the possible mechanism of the GBAP1/miR-22-3p/BMPR1A axis in HCC development. Furthermore, METTL3 strengthens the stability of GBAP1 in HCC cells via increasing the m^6^A modification level of GBAP1. Thus, GBAP1 was a potential tumor biomarker, prognosis indicator and therapeutic target in HCC.

## Supplementary Information

**Additional file 1.** Supplementary figures and methods.

**Additional file 2.** Differentially expressed lncRNAs in METTL3-ovexpressed Hep3B cells.

## Data Availability

The original contributions presented in the study are included in the article/Additional files. Further inquiries can be directed to the corresponding author.
