# Correction: METTL3-induced lncRNA GBAP1 promotes hepatocellular carcinoma progression by activating BMP/SMAD pathway

**DOI:** 10.1186/s13062-025-00673-4

**Published:** 2025-07-10

**Authors:** Runkun Liu, Guozhi Yin, Hang Tuo, Yixian Guo, Yifeng Zhu, Lei Zhang, Wei Yang, Qingguang Liu, Yufeng Wang

**Affiliations:** 1 Department of Hepatobiliary Surgery, The First Affiliated Hospital of Xi’an Jiaotong University, Xi’an, 710061 Shaanxi China; 2 Department of Geriatric Surgery, The First Affiliated Hospital of Xi’an Jiaotong University, Xi’an, 710061 Shaanxi China

**Correction: Liu et al. Biology Direct (2023) 18:53**

10.1186/s13062-023-00409-2

After publication of this article, the authors identified an error which unintendedly occured during assembling the Fig. 8D.

The incorrect Fig. 8:

**Fig. 8:**
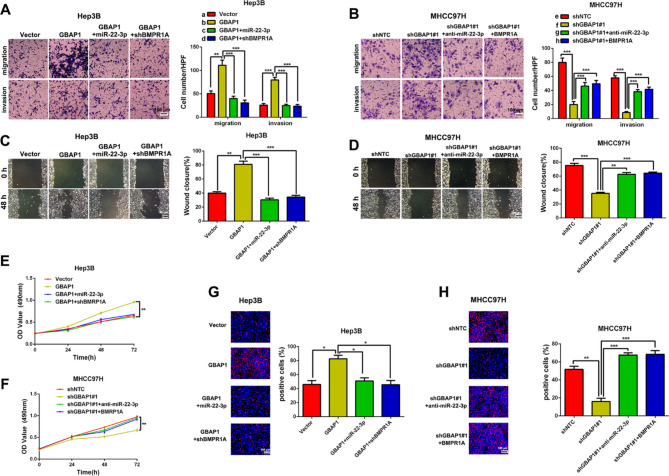
GBAP1 activates BMP/SMAD signaling under the mediation of miR-22-3p in HCC cells. **A** Transwell assay revealed that pcDNA/GBAP1 alone promoted migration and invasion of Hep3B cells, while miR-22-3p mimics or BMPR1A shRNA reversed the promotion effects of pcDNA/GBAP1 on migration and invasion of Hep3B cells. **P < 0.01 (Student’s t test). ***P < 0.001 (two-way ANOVA). **B** Transwell assay revealed that shGBAP1 alone inhibited migration and invasion of MHCC97H cells, while miR-22-3p inhibitors or pcDNA/BMPR1A reversed the inhibitory effects of shGBAP1 on migration and invasion of MHCC97H cells. ***P < 0.001 (Student’s t test). ***P < 0.001 (two-way ANOVA). **C** Wound healing assay revealed that pcDNA/GBAP1 alone promoted mobility of Hep3B cells, while miR-22-3p mimics or BMPR1A shRNA reversed the promotion effects of pcDNA/GBAP1 on Hep3B cells mobility. **P < 0.01 (Student’s t test). ***P < 0.001 (two-way ANOVA). **D** Wound healing assay revealed that shGBAP1 alone inhibited mobility of MHCC97H cells, while miR-22-3p inhibitors or pcDNA/BMPR1A reversed the inhibitory effects of shGBAP1 on MHCC97H cells mobility. ***P < 0.001 (Student’s t test). **P < 0.01, ***P < 0.001 (two-way ANOVA). **E** MTT assay revealed that pcDNA/GBAP1 alone promoted viability of Hep3B cells, while miR-22-3p mimics or BMPR1A shRNA reversed the promotion effects of pcDNA/GBAP1 on Hep3B cells viability. **P < 0.01 (two-way ANOVA with Sidak’s t test). **F** MTT assay revealed that shGBAP1 alone inhibited viability of MHCC97H cells, while miR-22-3p inhibitors or pcDNA/BMPR1A reversed the inhibitory effects of shGBAP1 on MHCC97H cells viability. **P < 0.01 (two-way ANOVA with Sidak’s t test). **G** EdU assay revealed that pcDNA/GBAP1 alone promoted Hep3B cells proliferation, while miR-22-3p mimics or BMPR1A shRNA reversed the promotion effects of pcDNA/GBAP1 on Hep3B cells proliferation. *P < 0.05 (Student’s t test). *P < 0.05 (two-way ANOVA). **H** EdU assay revealed that shGBAP1 alone inhibited MHCC97H cells proliferation, while miR-22-3p inhibitors or pcDNA/BMPR1A reversed the inhibitory effects of shGBAP1 on MHCC97H cells proliferation. **P < 0.01 (Student’s t test). ***P < 0.001 (two-way ANOVA). Magnification: 200 ×

The correct Fig. 8:

**Fig. 8:**
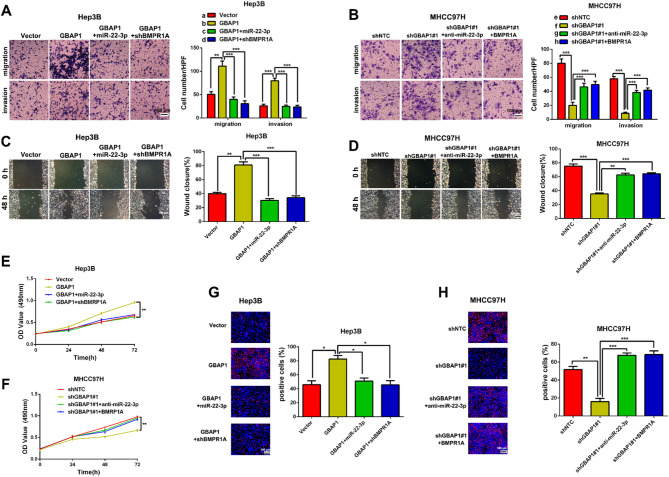
GBAP1 activates BMP/SMAD signaling under the mediation of miR-22-3p in HCC cells. **A** Transwell assay revealed that pcDNA/GBAP1 alone promoted migration and invasion of Hep3B cells, while miR-22-3p mimics or BMPR1A shRNA reversed the promotion effects of pcDNA/GBAP1 on migration and invasion of Hep3B cells. **P < 0.01 (Student’s t test). ***P < 0.001 (two-way ANOVA). **B** Transwell assay revealed that shGBAP1 alone inhibited migration and invasion of MHCC97H cells, while miR-22-3p inhibitors or pcDNA/BMPR1A reversed the inhibitory effects of shGBAP1 on migration and invasion of MHCC97H cells. ***P < 0.001 (Student’s t test). ***P < 0.001 (two-way ANOVA). **C** Wound healing assay revealed that pcDNA/GBAP1 alone promoted mobility of Hep3B cells, while miR-22-3p mimics or BMPR1A shRNA reversed the promotion effects of pcDNA/GBAP1 on Hep3B cells mobility. **P < 0.01 (Student’s t test). ***P < 0.001 (two-way ANOVA). **D** Wound healing assay revealed that shGBAP1 alone inhibited mobility of MHCC97H cells, while miR-22-3p inhibitors or pcDNA/BMPR1A reversed the inhibitory effects of shGBAP1 on MHCC97H cells mobility. ***P < 0.001 (Student’s t test). **P < 0.01, ***P < 0.001 (two-way ANOVA). **E** MTT assay revealed that pcDNA/GBAP1 alone promoted viability of Hep3B cells, while miR-22-3p mimics or BMPR1A shRNA reversed the promotion effects of pcDNA/GBAP1 on Hep3B cells viability. **P < 0.01 (two-way ANOVA with Sidak’s t test). **F** MTT assay revealed that shGBAP1 alone inhibited viability of MHCC97H cells, while miR-22-3p inhibitors or pcDNA/BMPR1A reversed the inhibitory effects of shGBAP1 on MHCC97H cells viability. **P < 0.01 (two-way ANOVA with Sidak’s t test). **G** EdU assay revealed that pcDNA/GBAP1 alone promoted Hep3B cells proliferation, while miR-22-3p mimics or BMPR1A shRNA reversed the promotion effects of pcDNA/GBAP1 on Hep3B cells proliferation. *P < 0.05 (Student’s t test). *P < 0.05 (two-way ANOVA). **H** EdU assay revealed that shGBAP1 alone inhibited MHCC97H cells proliferation, while miR-22-3p inhibitors or pcDNA/BMPR1A reversed the inhibitory effects of shGBAP1 on MHCC97H cells proliferation. **P < 0.01 (Student’s t test). ***P < 0.001 (two-way ANOVA). Magnification: 200 ×

The original publication has been corrected.

