# Supplementary material for: METTL3-induced lncRNA GBAP1 promotes hepatocellular carcinoma progression by activating BMP/SMAD pathway
Source: Biol Direct. 2023 Sep 1;18:53. doi: 10.1186/s13062-023-00409-2 (PMC10472583; doi:10.1186/s13062-023-00409-2)
Supplement: Supplementary file 1 — Additional file 1. Supplementary figures and methods. [file 13062_2023_409_MOESM1_ESM.docx]

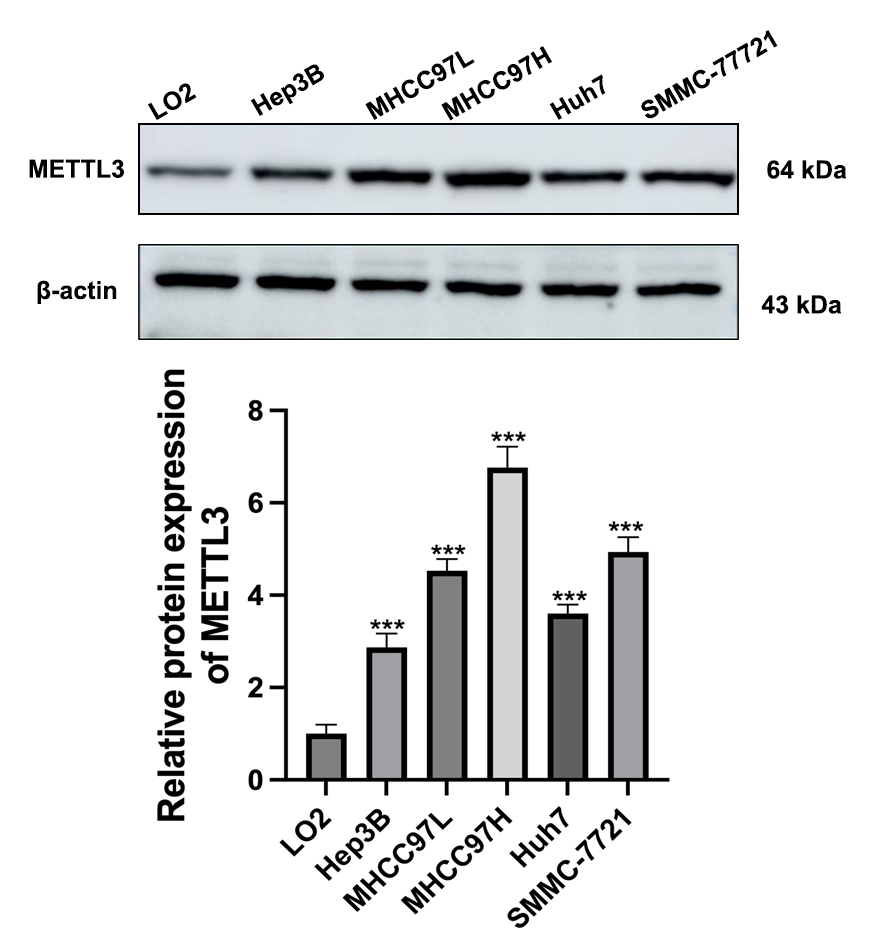


**Fig.S1** METTL3 protein expression was upregulated in HCC cell lines. Western blot was used to measure the METTL3 protein expression in HCC cell lines. The blots were quantized by Image J software. ****P* < 0.001 versus LO2 (two-way ANOVA).


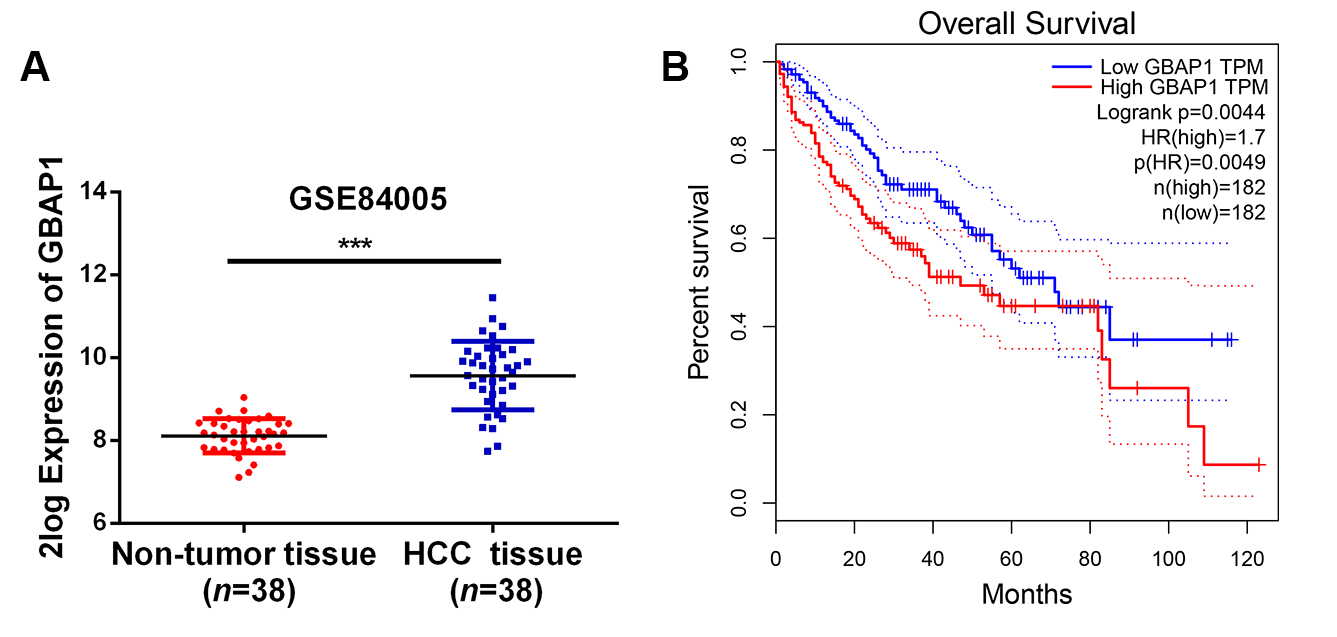


**Fig.S2** The expression and effect on HCC prognosis of GBAP1 in public dataset. **A** GEO dataset GSE84005 was used to measure GBAP1 expression in HCC. ****P* < 0.001 (Student’s t test). **B** The effect of GBAP1 on overall survival of HCC patients was analyzed by GEPIA platform.


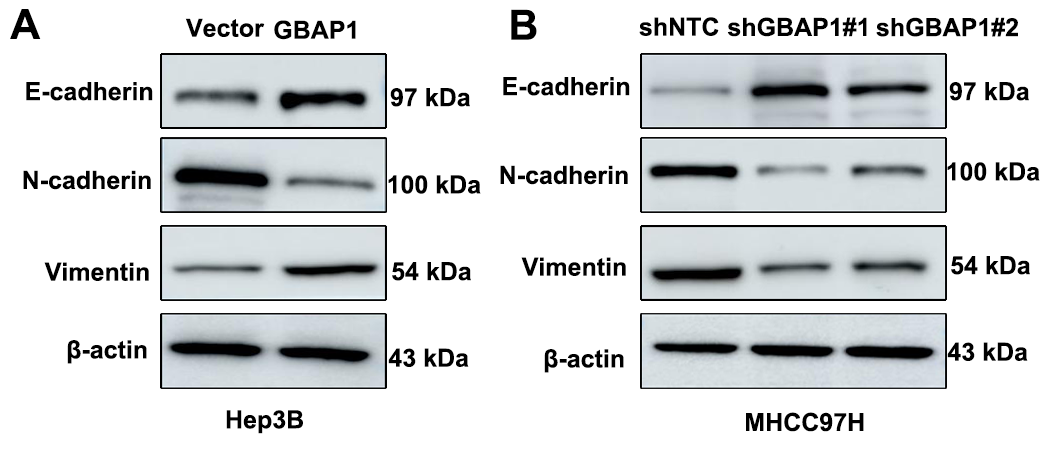


**Fig.S3** GBAP1 promotes EMT of HCC cells. **A** Western blot was applied to test EMT markers expression change in GBAP1 overexpressing subclones of Hep3B. B Western blot was applied to test EMT markers expression change in GBAP1 knockdown subclones of MHCC97H.


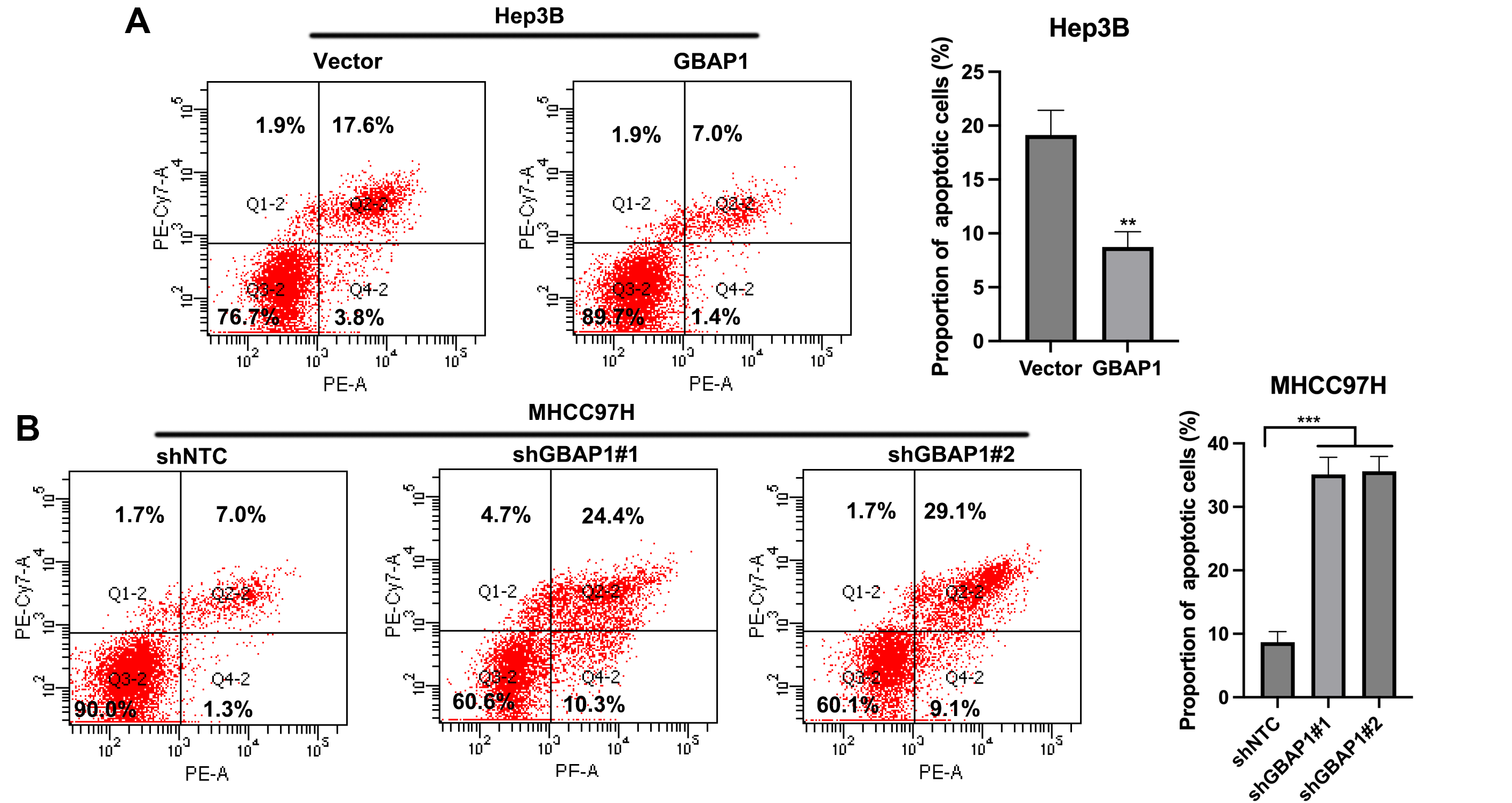


**Fig.S4** GBAP1 inhibits apoptosis of HCC cells. **A** Flow cytometry for detection of cell apoptosis was applied to test the cell apoptosis in overexpressing subclones of Hep3B. ****P* < 0.001 (Student’s t test). **B** Flow cytometry for detection of cell apoptosis was applied to test the cell apoptosis in GBAP1 knockdown subclones of MHCC97H. ****P* < 0.001 versus shNTC (two-way ANOVA).


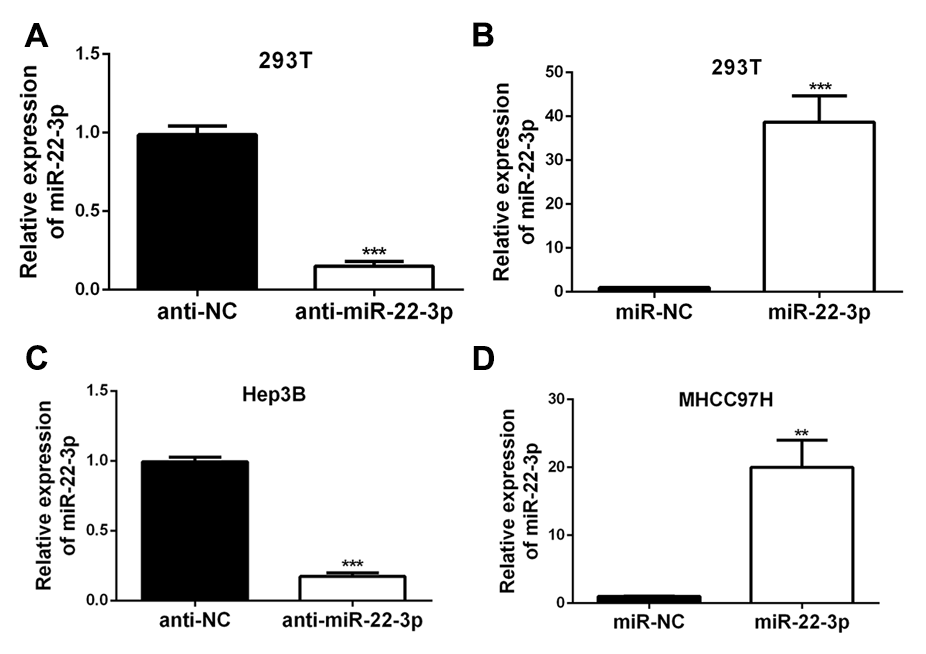


**Fig.S5** The efficiencies of miR-22-3p inhibitors and mimics in 293T and HCC cells. **A** RT-qPCR was performed to test the expression of miR-22-3p in 293T cell transfected with miR-22-3p inhibitors (anti-miR-22-3p) or control (anti-NC). **B** RT-qPCR was performed to test the expression of miR-22-3p in 293T cells transfected with miR-22-3p mimics (miR-22-3p) or control (miR-NC). **C** RT-qPCR was performed to test the expression of miR-22-3p in Hep3B cells transfected with miR-22-3p inhibitors (anti-miR-22-3p) or control (anti-NC). **D** RT-qPCR was performed to test the expression of miR-22-3p in 293T cells transfected with miR-22-3p mimics (miR-22-3p) or control (miR-NC). ***P*<0.01, ****P*<0.001


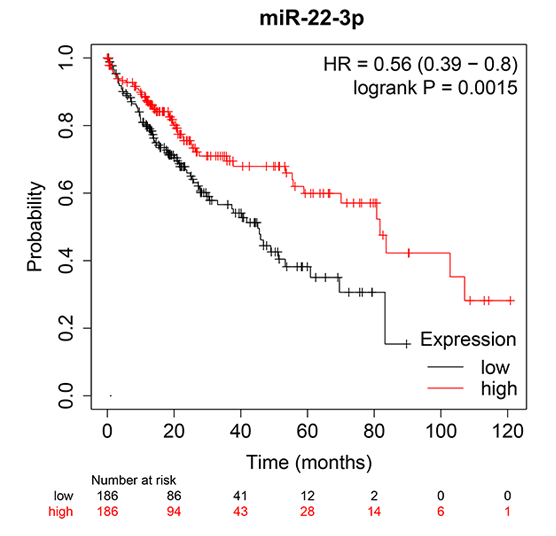


**Fig.S6** MiR-22-3p expression is associated with HCC patient prognosis. Data from platform Online Kaplan-Meier Plotter showed that HCC patients with lower miR-22-3p expression had worse prognosis than that with high miR-22-3p.


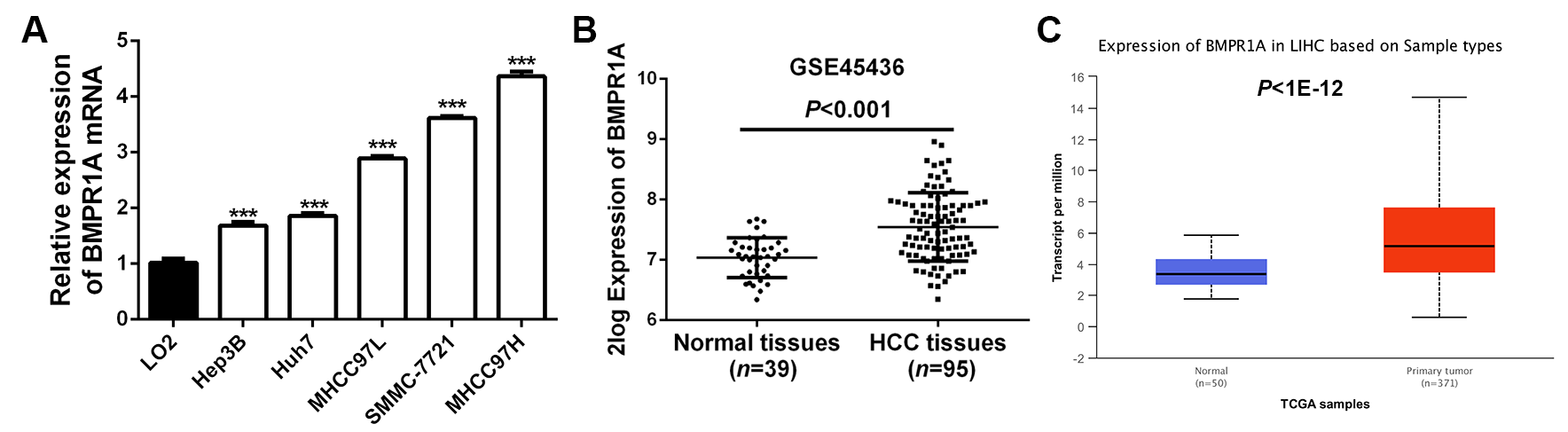


**Fig.S7** The expression of BMPR1A is significantly increased in HCC. **A** RT-qPCR analysis was performed to test BMPR1A mRNA expression in HCC cell lines and normal hepatic cell LO2. ****P*<0.001 (Student’s t test). **B** Data from GSE45436 showed that BMPR1A was significantly increased in HCC tissues (*n*=95) compared to normal tissues (*n*=39). ****P*<0.001 (Student’s t test). **C** Data from TCGA showed that BMPR1A expression was significantly increased in HCC tissues (*n*=371) compared to normal tissues (*n*=50).


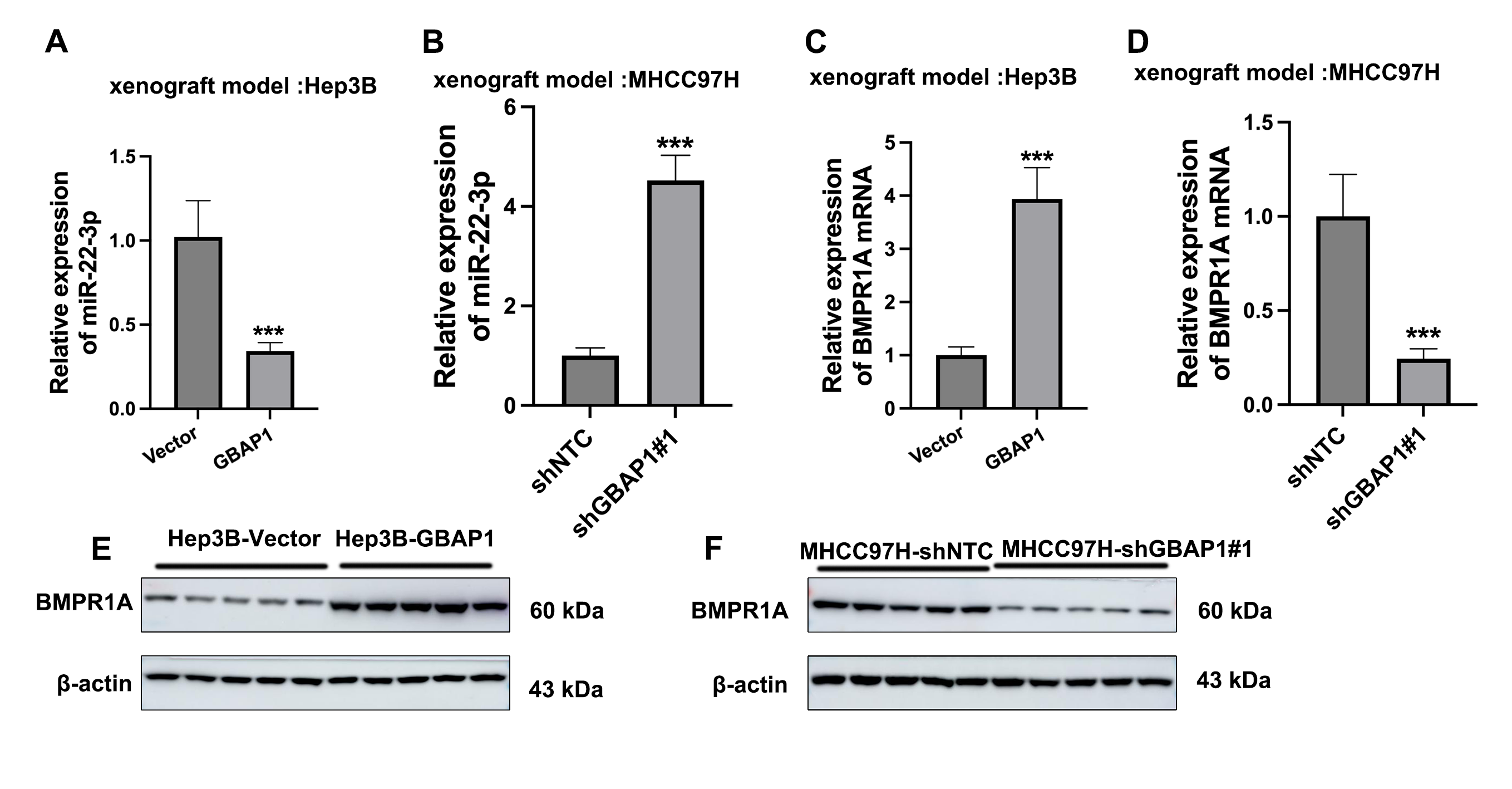


**Fig.S8** The effect of GBAP1 on miR-22-3p and BMPR1A expression in vivo. **A-D** RT-qPCR was applied to test the expression changes of miR-22-3p and BMPR1A mRNA in the corresponding tumor nodules. ****P* < 0.001 (Student’s t test). **E** and **F** Western blot was applied to test the expression changes of BMPR1A protein in the corresponding tumor nodules.


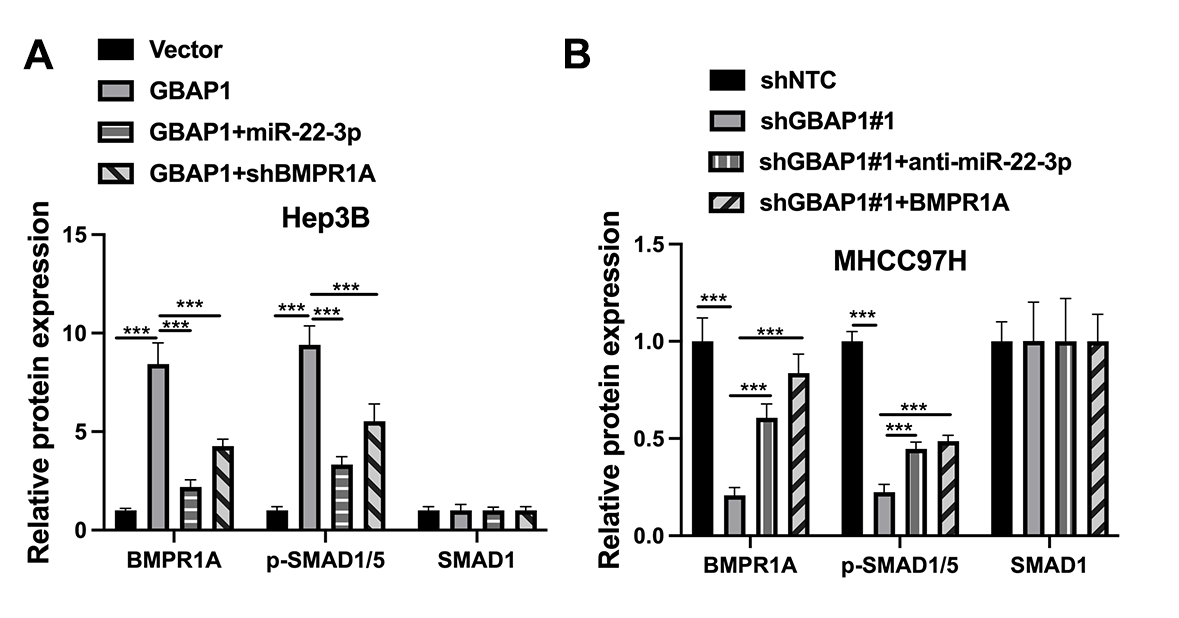


**Fig.S9** Quantification of Western blot in rescue experiments. **A** and **B** The corresponding blots were quantized by Image J software. ****P* < 0.001 (Student’s t test or two-way ANOVA).

**Materials and methods**

**Cell apoptosis assay**

The corresponding subclones cells (6 × 10^5^/well) were plated in 6 well plates. Then, cells were digested with pancreatin and washed with PBS 24 h after cell attachment. Flow cytometry for cell apoptosis was conducted by PE Annexin V Apoptosis Detection Kit I (#559763, Becton Dickinson bioscience, San Jose, CA, USA) according to the manufacturer’s protocols. Finally, flow cytometry was used for determining the apoptosis distribution.
